# Genuine Memory Deficits as Assessed by the Free and Cued Selective Reminding Test (FCSRT) in the Behavioural Variant of Frontotemporal Dementia. A Systematic Review and Meta-analysis Study

**DOI:** 10.1007/s11065-023-09613-3

**Published:** 2023-09-22

**Authors:** Luigi Macchitella, Giorgia Tosi, Francesco Giaquinto, Marika Iaia, Ezia Rizzi, Ylenia Chiarello, Maxime Bertoux, Paola Angelelli, Daniele Luigi Romano

**Affiliations:** 1 Department of Human and Social Sciences, University of Salento, Piazza Tancredi 7, 73100 Lecce, Italy; 2 Scientific Institute I.R.C.C.S. “E. Medea”, Unit for Severe Disabilities in Developmental Age and Young Adults (Developmental Neurology and Neurorehabilitation), Piazza Di Summa, 72100 Brindisi, Italy; 3 Department of Psychology, University of Milano—Bicocca, Piazza Ateneo Nuovo 1, 20126 Milan, Italy; 4 Univ. Lille, Inserm, CHU Lille, Lille Neuroscience & Cognition, LiCEND, DISTALZ, 42 rue Paul Duez, 59000 Lille, France

**Keywords:** Behavioural variant, Frontotemporal dementia, FCRST, Memory test, Meta-analysis

## Abstract

The current diagnostic criteria for the behavioural variant of frontotemporal dementia (bvFTD) foresee a relative sparing of long-term memory. Although bvFTD patients were thought to report secondary memory deficits associated with prefrontal dysfunctions, some studies indicated the presence of a “genuine memory deficit” related to mesial temporal lobe dysfunctions. Among various neuropsychological tests, the Free and Cue Selective Reminding Test (FCSRT) has been recommended to distinguish genuine from apparent amnesia. We conducted a systematic review and a random effect Bayesian meta-analysis to evaluate the nature and severity of memory deficit in bvFTD. Our objective was to determine whether the existing literature offers evidence of genuine or apparent amnesia in patients with bvFTD, as assessed via the FCSRT. On 06/19/2021, we conducted a search across four databases (PMC, Scopus, Web of Science, and PubMed). We included all studies that evaluated memory performance using the FCSRT in patients with bvFTD, as long as they also included either cognitively unimpaired participants or AD groups. We tested publication bias through the Funnel plot and Egger’s test. To assess the quality of studies, we used the Newcastle-Ottawa quality assessment scale adapted for cross-sectional studies. We included 16 studies in the meta-analysis. The results showed that bvFTD patients perform better than AD patients (pooled effects between 0.95 and 1.14), as their memory performance stands between AD and control groups (pooled effects between − 2.19 and − 1.25). Moreover, patients with bvFTD present both genuine and secondary memory disorders. As a major limitation of this study, due to our adoption of a rigorous methodology and stringent inclusion criteria, we ended up with just 16 studies. Nonetheless, our robust findings can contribute to the ongoing discussion on international consensus criteria for bvFTD and the selection of appropriate neuropsychological tools to facilitate the differential diagnosis between AD and bvFTD.

**Supplementary Information:**

The online version contains supplementary material available at 10.1007/s11065-023-09613-3.

## Introduction

Behavioural and socio-cognitive symptoms characterize the behavioural variant of frontotemporal dementia (bvFTD). When considering past and current diagnostic criteria, the neuropsychological profile includes deficits in executive functions with relatively preserved episodic memory (Neary et al., 1998; Rascovsky et al., 2011). Therefore, severe amnesia is not considered a clinical feature of bvFTD. On the contrary, it could be regarded as an exclusion criterion (Ahmed et al., 2021). Notably, the spared long-term memory has been presented as one key element to clinically distinguish bvFTD from Alzheimer’s disease (AD) (Dubois et al., 2007). However, the possibility that patients with bvFTD may suffer from memory disorders has been increasingly discussed over the last decade (see Hornberger & Piguet, 2012 for a historical perspective). When using word-list memory assessment through free recall procedures, patients in the early stage of bvFTD may show memory deficits that can be as severe as those observed in patients with AD (Hornberger et al., 2010; Pennington et al., 2011; Irish et al., 2014; for review, see Hornberger & Piguet, 2012; Ahmed et al., 2021). Such memory impairment has been supported by the meta-analysis conducted by Poos et al. (2018). Consequently, it is increasingly envisaged that severe amnesia should not preclude a diagnosis of bvFTD (Irish et al., 2014), as a complete absence of long-term memory deficits in bvFTD is unlikely (Hornberger & Piguet, 2012).

To reconcile the divergent views, it has been early suggested that long-term memory deficits exhibited by patients with bvFTD do not reflect “genuine amnesia” (or true amnesia) (see, e.g. Neary et al., 1998; Dubois et al., 2007; Collette et al., 2010; Pennington et al., 2011; Frisch et al., 2013). “Genuine amnesia” refers to an amnesic syndrome that characterizes patients with AD and is conceptualized as reflecting (being associated with) medial temporal lobe atrophy (Dubois et al., 2007, 2010; Pasquier et al., 2001). This type of amnesia implies impairment of the long-term memory storage and consolidation processes due to damaged medial temporal structures, including the hippocampus and adjacent structures (Dubois et al., 2007, 2010; Grober et al., 1988; Sarazin et al., 2010). Such impairment is typically observed in individuals with typical AD. In contrast, the memory difficulties presented by bvFTD patients were believed to be indicative of “apparent amnesia”. Apparent amnesia would be secondary to defective information encoding and/or retrieval strategies due to prefrontal cortex dysfunctions (see, e.g. Neary et al., 1998; Thomas-Anterion et al., 2000; Glosser et al., 2002; Collette et al., 2010; Pennington et al., 2011; Frisch et al., 2013; Lemos et al., 2014). For evidence and details concerning the involvement of the prefrontal cortex in long-term memory processes, see Simons and Spiers (2003) and Blumenfeld and Ranganath (2007). Following the distinction mentioned above, which is poorly supported by empirical (e.g. anatomical) evidence, a commonly shared hypothesis in the field is that the poor memory performance exhibited by bvFTD and AD patients reflects different neurocognitive deficits (Pennington et al., 2011; Frisch et al., 2013; see also Dubois et al., 2007). However, recent evidence has demonstrated similar hippocampal atrophy in bvFTD and AD patients (De Souza et al., 2013; Hornberger et al., 2012; Mansoor et al., 2015), suggesting that damage in medial temporal structures observed in bvFTD could also account for the amnesia experienced by those patients (Cerami et al., 2016; Irish et al., 2014).

Beyond free recall-based procedures, specific neuropsychological tests have been developed to assist in distinguishing genuine “AD-like” amnesia from apparent “bvFTD-like” amnesia. One critical test that exemplifies this feature is the Free and Cue Selective Reminding Test (FCSRT) (Grober & Buschke, 1987; Grober et al., 1988). It has been suggested that the FCSRT enables the discrimination between “genuine” storage and consolidation memory impairments from “apparent” memory disorders secondary to (resulting from) encoding and retrieval deficits (Dubois et al., 2008; Grober et al., 1988; Sarazin et al., 2010). Consequently, the FCSRT has been recommended for differential diagnosis of various forms of dementia, including AD and FTD (Boccardi et al., 2021; Costa et al., 2017; Dubois et al., 2007, 2010; Sorbi et al., 2012). The key feature of the FCSRT is the use of semantic categories to support and control effective information encoding and facilitate the retrieval of stored information during recall (for details concerning the FCSRT's procedures, see Buschke, 1984; Grober & Buschke, 1987; Grober et al., 1988; Sarazin et al., 2007).

Notably, studies utilizing the FCSRT have reported that bvFTD patients might exhibit impairment in storage and consolidation processes (Bertoux et al., 2014) and that these memory deficits cannot be solely attributed to by executive dysfunctions (Bertoux et al., 2016a). Instead, they may reflect damage to medial temporal cerebral structures (Bertoux et al., 2018; Fernández-Matarrubia et al., 2017).

In summary, there is substantial evidence indicating that patients with bvFTD may exhibit low performance in memory tasks (Hornberger & Piguet, 2012; Poos et al., 2018); however, the underlying neurocognitive impairment responsible for these memory difficulties has not yet been clearly elucidated.

The present study aimed to assess the nature (genuine vs apparent) and severity of the memory deficits characterizing the bvFTD. To achieve this objective, we conducted a systematic review and a meta-analysis focused on studies that directly compared the memory performance using the FCSRT between patients with bvFTD and cognitively unimpaired participants (UP) or AD patients.

Previous reviews and meta-analyses have explored the presence of memory impairments in patients with bvFTD (Hornberger & Piguet, 2012; Hutchinson & Mathias, 2007; Poos et al., 2018). However, none of them have specifically investigated the nature of such a deficit. While it is widely acknowledged that bvFTD patients exhibit impaired performance on memory tests, whether this represents a genuine or secondary deficit has not been previously examined at the meta-analytic level. Furthermore, in this study, we aimed to investigate the severity of memory deficits compared to cognitively unimpaired participants and patients with AD from a meta-analytic standpoint for the first time.

This perspective represents a novel approach from a meta-analytic viewpoint as it allows to examine the international recommendation endorsing the use of the FCSRT. Additionally, it addresses a common clinical practice concerning the neuropsychological differentiation between AD and FTD, and it raises questions regarding the existing diagnostic criteria for bvFTD.

## Materials and Methods

### Identification of Studies

The study is pre-registered in PROSPERO (CRD42021265945). The literature review was conducted following the Preferred Reporting Items for Systematic Reviews and Meta-Analyses (PRISMA) guidelines (Page et al., 2021). On 06/19/2021, we performed a comprehensive search across four databases (PMC, Scopus, Web of Science, and PubMed) using the following search string: (“frontotemporal dementia” OR “frontal dementia” OR “Pick’s disease” OR “frontotemporal lobe dementia” OR “frontal lobe dementia” OR “dementia of the frontal type” OR “behavioral variant frontotemporal dementia “OR “bvFTD”) AND (“Free and Cued Selective Reminding Test “OR “FCSRT” OR “Grober” OR “Buschke”). To identify and remove potential duplicates from the retrieved records, we utilized the R package “Revtools” screening for identical titles or DOIs. Titles and abstracts of the remaining records were thoroughly reviewed, and potentially eligible papers were collected in full text. Each abstract was independently analysed by two authors, and in cases of disagreement, the author team collectively reviewed the record.

It should be noted that we followed the same procedure for the studies that used the California Verbal Learning Test, a test that, similarly to the FCSRT, (i) shares the critical feature of having a cued recall phase and (ii) has been commonly employed in the field of AD/FTD to examine memory deficits. We identified 14 papers potentially useful that used the CVLT. However, none of these studies reported the necessary information required for our analyses, and despite multiple attempts to contact the authors, they did not provide the requested data, specifically the subtest scores. Therefore, the methodology regarding this particular effort is presented in the Supplementary Material, and the following sections will solely focus on the part of the study that pertains to the FCSRT.

### Study Selection

The meta-analysis included all English-language studies that examined memory performance in patients with bvFTD using the FCSRT. We decided to include only articles that met the following criteria: (i) assessment of memory performance in both a bvFTD patient group and a cognitively unimpaired participant or an AD patient group, (ii) a sample size greater than 10 for each group to ensure reliable effect sizes, and (ii) confirmation of bvFTD diagnosis using the diagnostic criteria of Neary et al. (1998) or Rascovsky et al. (2011).

### Data Extraction

Each eligible full-text article was independently analysed by two reviewers of the authors’ team to extract the following subscores in the FCSRT: Encoding (ENC, i.e. verbal encoding phase), Free ImmediateRecall (FIR, i.e. free learning phase), Cue ImmediateRecall (CIR, i.e. semantically cued learning phase), Total Immediate (TIR, i.e. free + semantically cued learning phase), Free Delayed Recall (FDR, i.e. free retrieval), Cue Delayed Recall (CDR, i.e. semantically cued retrieval), Total Delayed Recall (TDR, i.e. free + semantically cued retrieval), Recognition (R, i.e. recognition between distractors), Index of Sensitivity of Cueing (ISC, i.e. facilitation role of the semantic cue). We also gathered the following data: bvFTD diagnostic criteria and type of diagnosis (possible, probable, definite, or unknown) (see Table 1). We applied the levels of diagnostic certainty proposed by Rascovsky et al. (2011) to classify the diagnosis type. Diagnosis of possible bvFTD is based solely on the clinical syndrome. In contrast, the diagnosis of probable bvFTD is based on the clinical syndrome plus demonstrable functional decline and imaging findings consistent with bvFTD. Finally, diagnosis of definitive bvFTD is limited to patients who exhibit the bvFTD clinical syndrome and who also have a pathogenic mutation or histopathological evidence of FTLD. The levels of diagnostic certainty proposed by Rascovsky et al. (2011) were considered non-applicable (NA; see Table 1) when (i) studies used diagnostic criteria proposed by Neary et al. (Neary et al., 1998) and (ii) studies did not make the level of diagnostic certainty explicit.

**Table 1:** Characteristics of the studies included in the meta-analysis

	*N*			*Age (yrs mean* ± *std deviation)*			*Gender (% female)*			*Education (yrs mean* ± *std deviation)*			*MMSE (mean* ± *std deviation)*			*Diagnostic criteria*	*Diagnosis*^c^	*Language*
	*FTD*	*AD*	*UP*	*FTD*	*AD*	*UP*	*FTD*	*AD*	*UP*	*FTD*	*AD*	*UP*	*FTD*	*AD*	*UP*			
Lage et al. (2021)	18	17	29	68.83 ± 8.71	68.17 ± 6.69	66.21 ± 5.51	22.22	66.67	79.31	NA	NA	NA	23.5 ± 2.73	16.72 ± 5.23	28.96 ± 0.92	Rascovsky et al. (2011)	NA	
Bertoux et al. (2020)	22	23		61.86 ± 9.57	69 ± 7.96		3	11		14.47 ± 4.81	14.2 ± 5.33		25.86 ± 2.91	24.91 ± 2.86		Mackenzie et al. (2009)^a^	Definitive	French
Pozueta et al. (2019)	20	32		72.7 ± 7.28	66.44 ± 5.92		25	71.87		10.6 ± 3.5	10.5 ± 3.33		23.44 ± 3.7	21.52 ± 5		Rascovsky et al. (2011)	NA	Spanish
Canu et al. (2017)	27	62	48	57.7 ± 8.1	59.7 ± 4.1	57.4 ± 6.3	40.74	59.68	64.58	12.3 ± 2.8	12 ± 2.6	13.3 ± 2.8	22.5 ± 6.2	14.9 ± 6.2	29.8 ± 0.6	Rascovsky et al. (2011)	NA	
Boutoleau-Bretonnière et al. (2015)	36	22		66 ± 8.3	65.4 ± 7.3		42	45		NA	NA		23.6 ± 3.6	23.5 ± 2.5		Rascovsky et al. (2011)	Possible	French
Matuszewski et al. (2006)	20		21	67.9 ± 9.1		69.85 ± 8.57	NA		NA	NA		NA	24.3 ± 3.96		NA	Neary et al. (1998)	NA	French
Piolino et al. (2003)	15	13	18	67.8 ± 9.4	73.1 ± 5.5	69.4 ± 2.9	NA	NA	NA	NA	NA	NA	25.6 ± 1.9	22.3 ± 2.1	NA	Neary et al. (1998)	NA	French
Alcolea et al. (2019)	96	527	300	68.78 ± 9.6	73.21 ± 6.97	59.35 ± 10.61	29.17	59.39	59	11.48 ± 4.7	10.73 ± 4.66	15.54 ± 4.31	26.01 ± 3.26	24.42 ± 3.44	29.2 ± 1	Rascovsky et al. (2011)	Possible, probable or definitive	
Cerciello et al. (2017)	9	15	20	72.3 ± 6.4	75.1 ± 5.3	73.3 ± 5.8	44.44	46.67	50	9.5 ± 3.2	7.5 ± 3.7	9.4 ± 3.8	21.3 ± 0.9	21.1 ± 0.7	26.9 ± 0.6	Neary et al. (1998)	NA	Italian
Lemos et al. (2014)	32	32	32	68.56 ± 1.19	69.72 ± 1.27	68.59 ± 1.27	31.25	46.88	31.25	6.97 ± 0.84	6.91 ± 0.87	7.06 ± 0.86	26.88 ± 0.43	21.22 ± 0.7	29.06 ± 0.2	Neary et al. (1998); Rascovsky et al. (2011)	NA	Portuguese
Teichmann et al. (2017)	69	200		67 ± 1	68.1 ± 0.6		46.38	59		11.4 ± 0.5	10.5 ± 0.3		21.6 ± 0.8	19.1 ± 0.5		Rascovsky et al. (2011)	NA	French
Bertoux et al. (2018)	15	34	29	68.27 ± 9.41	74.11 ± 6.7	71.72 ± 5.8	40	NA	NA	13.67 ± 7.04	10.79 ± 4.8	12.86 ± 4	24.67 ± 4.42	21 ± 4.7	28.28 ± 1.5	Rascovsky et al. (2011)^b^	Probable	French
Fernandez-Matarrubia et al. (2017)	26	29	24	71.3 ± 8.08	75.3 ± 7.6	67.4 ± 11.1	38.46	75.86	54.17	7.5 ± 4.45	8.1 ± 5.1	9.3 ± 3.9	24.47 ± 10.55	22.93 ± 4.28	27.54 ± 2.64	Rascovsky et al. (2011)	Probable	Spanish
Bertoux et al. (2014)	44	56	22	66.9 ± 8.3	66.4 ± 9.4	66.7 ± 9.3	43.18	57.14	40.91	10.8 ± 3.9	12.3 ± 3.5	12.8 ± 2.4	23.1 ± 3.6	21.9 ± 4.4	29 ± 2.6	Rascovsky et al. (2011)^b^	NA	French
Basely et al. (2013)	25	85		66.6 ± 11	72 ± 9		40	64.71		NA	NA		20.9 ± 4.4	17.7 ± 6.1		Neary et al. (1998)	NA	
Bertoux et al. (2016a)	39		60	64.15 ± 15.47		68.78 ± 7.05	51.28		NA	11.44 ± 4.02		12.81 ± 3.04	24.6 ± 4.41		29.22 ± 0.93	Rascovsky et al. (2011)^b^	NA	French

*FTD* behavioural variant of frontotemporal dementia, *AD* Alzheimer’s disease, *UP* cognitively unimpaired participants

^a^The paper includes bvFTD confirmed post-mortem after a pathophysiological examination

^b^Rascovsky criteria were followed except for the neuropsychology criterion 6

^c^*Definitive diagnosis* includes patients with pathophysiological or genetic confirmation

**Fig. 1:**
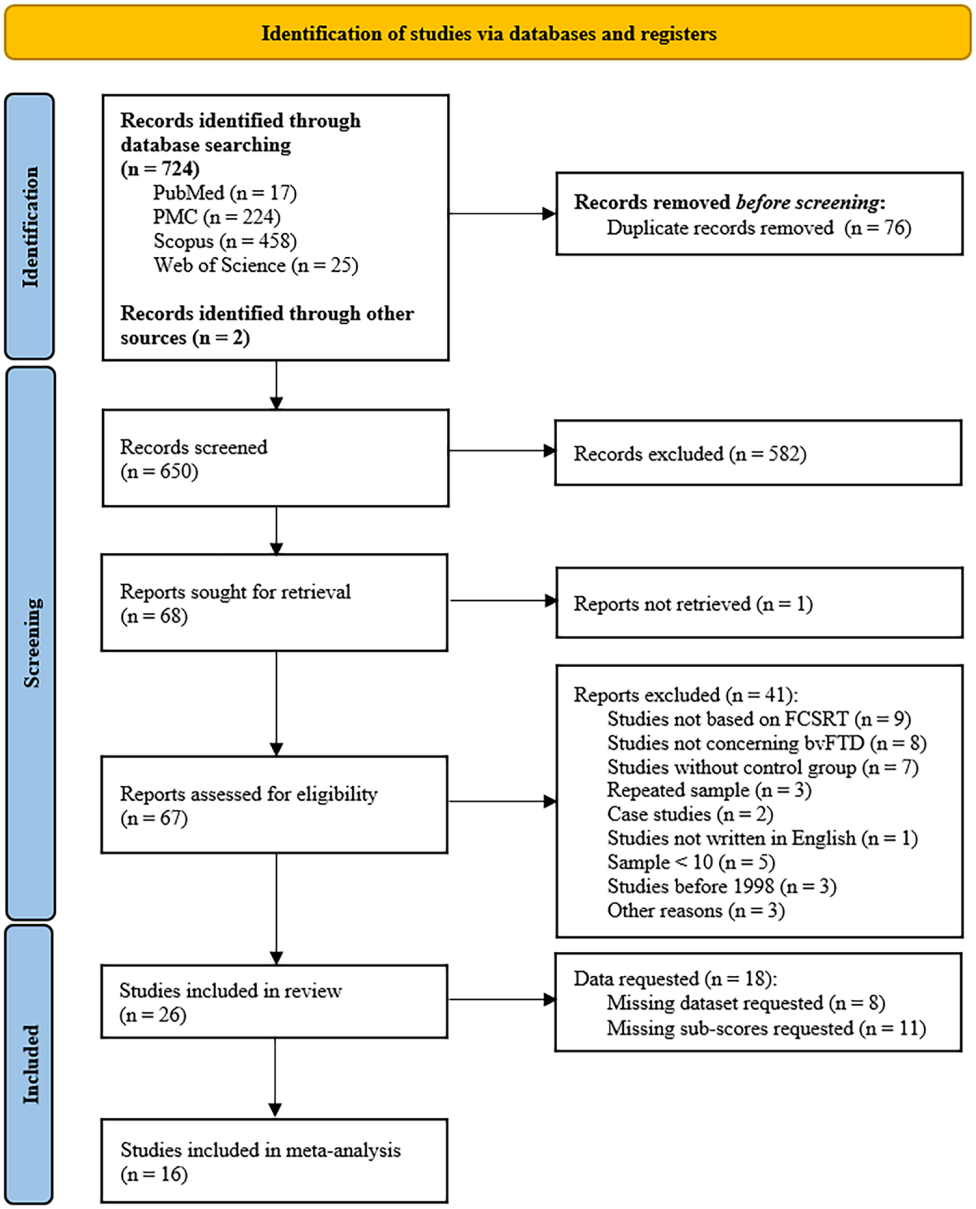
PRISMA 2020 flow diagram for new systematic reviews included searches of databases and registers only (Page et al., 2021). The figure shows the inclusion process of eligible studies and the reasons for the exclusion. Two studies were excluded because they had a sample size of three, for which estimating effect size was unreliable

When the subtype of FTD was not defined, or the raw test scores were not available for the patients and at least one of the control groups, we contacted the corresponding author for the missing data. We reached out to 15 authors for additional information for 17 papers. We made three attempts before excluding the paper. Out of the authors contacted, nine authors responded, and two provided numerical data. In cases where multiple studies referred to the same cohort of patients, we asked the original authors to clarify any overlapping participants. If disambiguation was not possible, we included only the data from the study with the largest sample from the same cohort. To illustrate the search procedure, we have included a PRISMA Flow Diagram (Page et al., 2021) in Fig. 1.

### Study Quality Assessment

To assess the quality of studies, we used a modified version of the Newcastle-Ottawa quality assessment scale adapted for cross-sectional studies (Modesti et al., 2016). Each paper was reviewed by one of the authors of the present work. We extracted the needed information from each study and assigned a rating based on the instructions reported in the Supplementary Materials. In cases of disagreement, the authors’ team reviewed the record to reach a consensus. The scale evaluates three main features: (i) the selection strategy (including representativeness of the sample, sample size, non-respondents, and ascertainment of exposure); (ii) the comparability of the samples (we considered impairment severity—as measured by MMSE and/or CDR—as the more important factor and either age, gender, or school-age as additional factors); and (iii) the outcome (including assessment of the outcome and statistical test). The total score on the Newcastle-Ottawa quality assessment scale ranges from 0 to 10, with a suggested interpretation by the original authors: good studies (7–10 stars), satisfactory studies (5–6 stars), and unsatisfactory studies (0–4 stars). However, in our specific case, one question was not applicable, so the actual range was 0–9. We thus suggest taking the total score cautiously, while readers may find interest in seeing each specific level of the quality assessment reported in Table 2. In the case of heterogeneity, the level of quality can be used as a threshold for the inclusion criteria or as a meta-regressor. However, as mentioned earlier, we did not observe heterogeneity and therefore did not use it in our analysis.

**Table 2:** Qualitative assessment checklist for observational studies

**Study**	**Selection (maximum 5 stars)**				**Comparability (maximum 2 stars)**				**Outcome (maximum 3 stars)**		**Total**
	**Representativeness**	**Sample size**	**Non-respondents**	**Exposure ascertainment**	**Severity (MMSE/CDR)**		**Additional factors**		**Assessment**	**Statistics**	
Lage et al. (2021)	1	0	NA	2	1	Matched	1	Controlled^abc^	2	1	8
Bertoux et al. (2020)	1	0	NA	2	1	Matched	1	Matched^ab^	2	1	8
Pozueta et al. (2019)	1	0	NA	2	1	Controlled	1	Controlled^abc^	2	1	8
Canu et al. (2017)	1	0	NA	2	0		1	Matched^b^	2	1	7
Boutoleau-Bretonnière et al. (2015)	1	1	NA	2	1	Matched	1	Matched^d^	0	1	7
Matuszewski et al. (2006)	0	0	NA	2	0		1	Matched^a^	0	0	3
Piolino et al. (2003)	0	0	NA	2	1	Matched	1	Matched^abc^	0	0	4
Alcolea et al. (2019)	1	1	NA	2	1	Controlled	1	Controlled^abc^	2	NA	8
Cerciello et al. (2017)	1	0	NA	2	1	Matched	1	Matched^abc^	2	1	8
Lemos et al. (2014)	1	0	NA	2	1	Matched	1	Matched^abc^	2	1	8
Teichmann et al. (2017)	1	0	NA	2	1	Matched	1	Matched^b^	1	1	7
Bertoux et al. (2018)	1	0	NA	2	0		1	Matched^abc^	1	1	6
Fernandez-Matarrubia et al. (2017)	0	0	NA	2	1	Matched	1	Matched^abc^	1	1	6
Bertoux et al. (2014)	1	0	NA	2	1	Matched	1	Matched^abc^	2	1	8
Basely et al. (2013)	1	0	NA	2	0		0		2	1	6
Bertoux et al. (2016a)	1	0	NA	2	1	Matched	1	Matched^abc^	2	1	8

The table reports the number of stars we assigned to each study in each category following the Newcastle-Ottawa quality assessment scale adapted for cross-sectional studies. Good studies: 7–10 stars, satisfactory studies: 5–6 stars, unsatisfactory studies: 0–4 stars

^a^Comparison between FTD and UP

^b^comparison between FTD and AD

^c^comparison between AD and HC

^d^comparison between FTD and AD, PSP, and BP patients as a whole

### Data Synthesis and Analysis

Effect sizes were calculated to determine the difference in test scores between (i) patients with bvFTD and healthy control participants and (ii) patients with bvFTD and AD. The test scores used for the analysis were Free Immediate Recall, Total Immediate Recall, Free Delayed Recall, and Total Delayed Recall. These specific subscores were considered crucial because the total score serves a proxy of genuine amnesia, while a potentially worse performance on the free score indicates the presence of unspecific memory impairments (i.e. genuine and secondary). It is worth noting that many studies have also focused selectively on these scores as well.

To estimate the effect size, we calculated Hedges’ g (the standardized difference between the groups) and its standard error using the R function “metacont” in the package “meta”. We chose Hedges’ g because it corrects for bias that may arise from small sample sizes (Hedges & Olkin, 1985). We employed a random-effects model for the analysis. Fitting a frequentist meta-analysis allowed us to check for the presence of outliers (by using the R function “find.outliers” in the package “dmetar”) and influential points (by using the R function “InfluenceAnalysis” in the package “dmetar”). The “find.outliers” function considers a study as an outlier if its confidence interval does not overlap with the confidence interval of the pooled effect. Studies with extreme effect sizes may distort the pooled effect estimate leading to between-study heterogeneity. Therefore, if extreme effect sizes were identified, we removed those studies from the analysis and reevaluated the pooled effect.

We further examined the potential presence of publication bias through the funnel plot and Egger’s test by using the R functions “funnel.meta” and “metabias” in the package “meta” (see Supplementary Material).

Then, we conducted a Bayesian random effect meta-analysis using the R function “brm” in the package “brms” to calculate the pooled effect size and the heterogeneity between studies. Bayesian models have several advantages both theoretically and practically. They allow for the incorporation of prior knowledge into the analysis, leading to more precise estimates. Bayesian statistics is a theoretical platform for updating information, thus allowing the inclusion of additional data as soon as they are available. This meta-analysis can also be seen as a first benchmark that can be further developed and updated in the future. In line with an Open Science perspective, we have made the data and analysis scripts publicly available (https://osf.io/eazck/?view_only=790c6423122a4583960bc9190167924b) to facilitate transparency and reproducibility.

The estimation started from a non-informative prior with a normal distribution (mean = 0, scale = 10) for the effect size and a half-Cauchy distribution (mean = 0, scale = 0.5) for the heterogeneity. We also checked for the stability of the results (i.e. prior robustness check) by trying different prior distributions (see Supplementary Materials). We conducted posterior predictive checks to assess the model convergence and the overall validity (see Supplementary Materials). The direction of the effect size was negative if the performance of the bvFTD patient group was worse than the control or AD patient group.

## Results

We included 16 studies in the meta-analysis (see Table 1 for studies’ characteristics). The results are reported based on the populations studied and the available FCSRT subscores.

### bvFTD vs Cognitively Unimpaired Participants

#### Free Immediate Recall

**Fig. 2:**
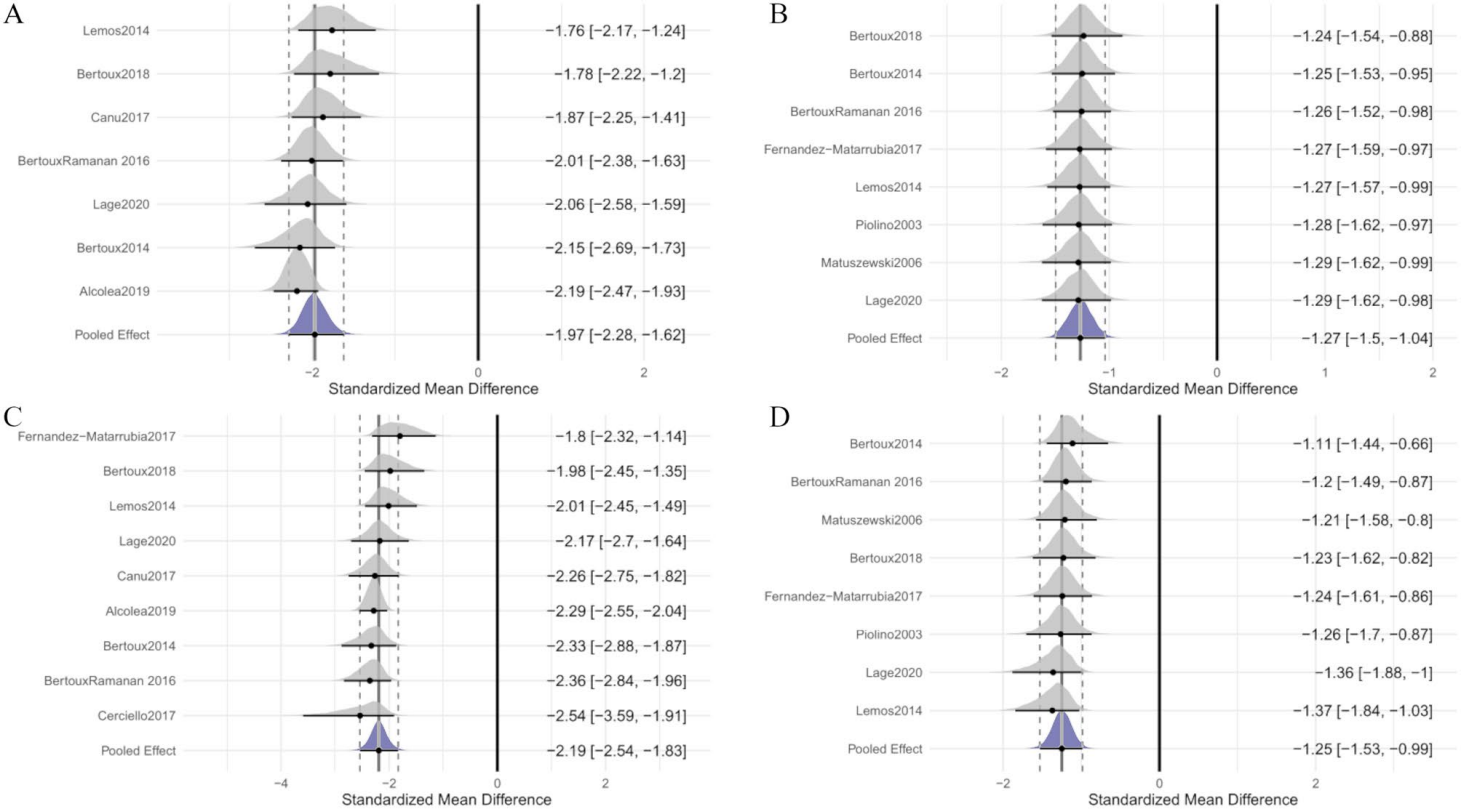
Forest plot illustrating effect sizes and 95% credible intervals for each study comparing bvFTD patients to healthy subjects (**A** Free Immediate Recall; **B** Total Immediate Recall; **C** Free Delayed Recall; **D** Total Delayed Recall). Negative values indicate worse performance for bvFTD than for cognitively unimpaired participants

A total of 271 patients with bvFTD and 520 cognitively unimpaired participants from 7 studies were included in the meta-analysis. No outliers or influential points were detected. The overall weighted effect size for patients versus cognitively unimpaired participants was − 1.98 (95% CI [− 2.30, − 1.65]); heterogeneity was tau = 0.29 (95% CI [0.03, 0.70]) (Fig. 2A), indicating that patients performed worse on Free Immediate Recall subtest compared to cognitively unimpaired participants, with a difference of approximately two standard deviations. Egger’s test for publication bias was not significant (bias: 1.94; *t*(5) =  − 1.34; *p* = 0.24), suggesting no evidence of publication bias.

#### Total Immediate Recall

A total of 209 patients with bvFTD and 253 cognitively unimpaired participants from 8 studies were included in the meta-analysis. The study by Alcolea et al. (2019) was excluded from the original set of 9 records due to being an outlier (see Supplementary Materials). The overall weighted effect size for patients versus cognitively unimpaired participants was − 1.27 (95% CI [− 1.50, − 1.04]); heterogeneity was tau = 0.11 (95% CI [0.00, 0.36]) (Fig. 2B). The estimated effect size indicates that patients performed worse on the Total Immediate Recall subtest compared to cognitively unimpaired participants, with a difference of slightly over one standard deviation. The Egger’s test for publication bias was not significant again (bias: − 1.03; *t*(6) =  − 1.12; *p* = 0.31).

#### Free Delayed Recall

A total of 306 patients with bvFTD and 564 cognitively unimpaired participants from 9 studies were included in the meta-analysis. No outliers were identified; however, the study by Fernandez-Matarrubia et al. (2017) was deemed influential. The overall weighted effect size for patients versus cognitively unimpaired participants was − 2.19 (95% CI [− 2.55, − 1.83]); heterogeneity was tau = 0.34 (95% CI [0.03, 0.84]) (Fig. 2C). Results indicate that patients performed significantly worse on Free Delayed Recall subtest compared to cognitively unimpaired participants. Egger’s test did not reveal any significant publication bias (bias: − 0.04; *t*(7) =  − 0.03; *p* = 0.98).

#### Total Delayed Recall

A total of 209 patients with bvFTD and 235 cognitively unimpaired participants from 8 studies were included in the meta-analysis. The studies by Alcolea et al. (2019) and Cerciello et al. (2017) were omitted from the original pull of 10 records as they were identified as outliers (see Supplementary Materials). The overall weighted effect size for patients versus cognitively unimpaired participants was − 1.25 (95% CI [–1.51, –0.98]); heterogeneity was tau = 0.19 (95% CI [0.01, 0.54]) (Fig. 2D), indicating that patients performed worse on Total Delayed Recall subtest compared to cognitively unimpaired participants. The Egger’s test did not reveal significant evidence of publication bias (bias: − 2.13; *t*(6) =  − 1.02; *p* = 0.35).

### bvFTD vs AD

#### Free Immediate Recall

**Fig. 3:**
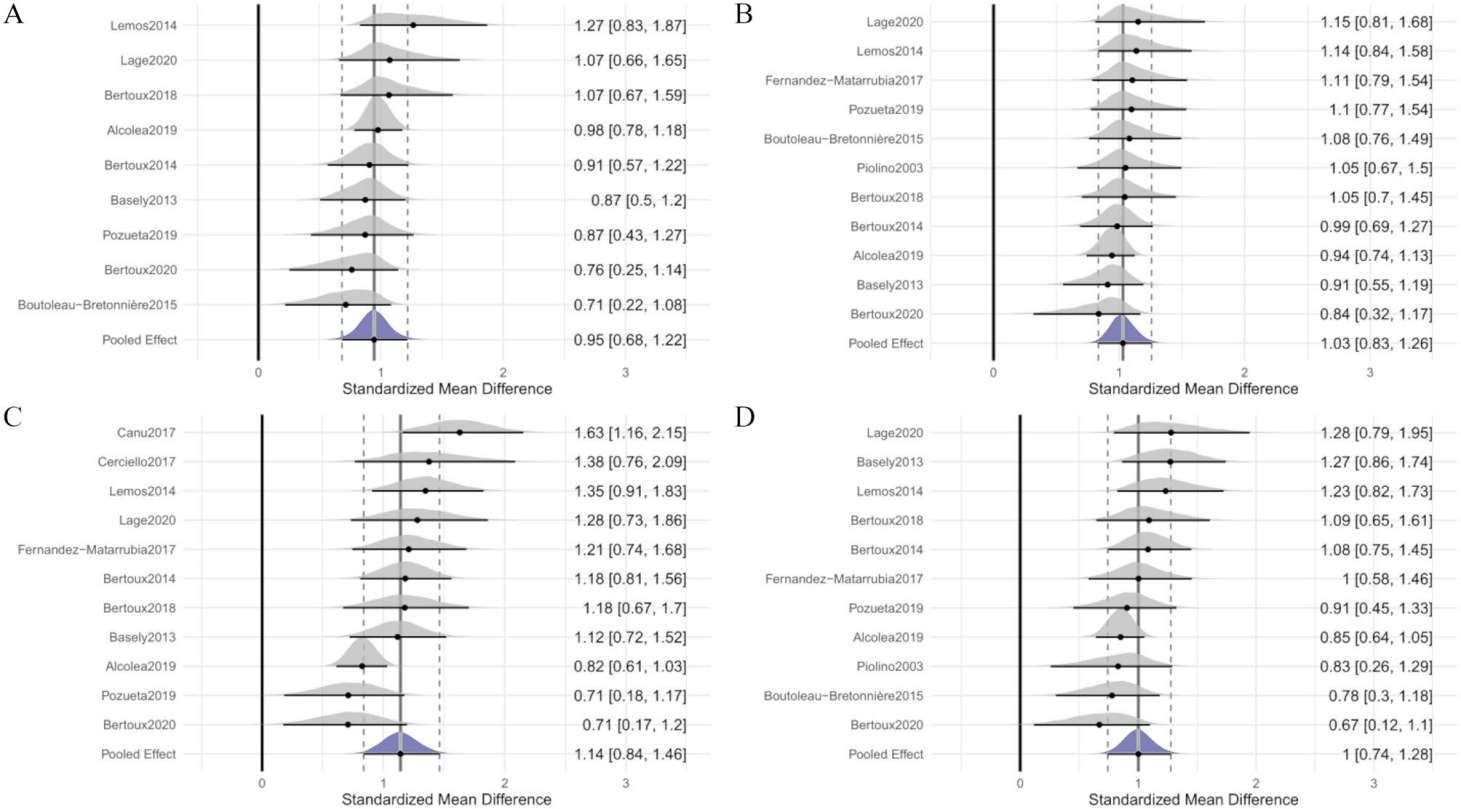
Forest plot illustrating effect sizes and 95% credible intervals for each study comparing bvFTD patients to AD patients (**A** Free Immediate Recall; **B** Total Immediate Recall; **C** Free Delayed Recall; **D** Total Delayed Recall). Positive values indicate worse performance for AD than for bvFTD

A total of 308 patients with bvFTD and 828 patients with AD from 9 studies were included in the meta-analysis. The studies by Teichmann et al. (2017) and Canu et al. (2017) were omitted from the original pull of 11 records as they were identified as outliers (see Supplementary Materials). The overall weighted effect size for bvFTD versus AD was 0.95 (95% CI [0.67, 1.23]); heterogeneity was tau = 0.95 (95% CI [0.68, 1.23]) (Fig. 3A), indicating that AD patients performed worse on Free Immediate Recall subtest compared to the bvFTD patients. The Egger’s test did not reveal significant evidence of publication bias (bias: 0.13; *t*(7) = 0.10; *p* = 0.92).

#### Total Immediate Recall

A total of 349 patients with bvFTD and 871 patients with AD from 11 studies were included in the meta-analysis. The study by Teichmann et al. (2017) was omitted from the original pull of 12 records due to being identified as an outlier (see Supplementary Materials). The overall weighted effect size for bvFTD versus AD was 1.03 (95% CI [0.84, 1.25]); heterogeneity was tau = 0.19 (95% CI [0.01, 0.47]) (Fig. 3B), indicating that AD patients performed worse on Total Immediate Recall subtest compared the bvFTD patients. The Egger’s test did not reveal significant evidence of publication bias (bias: 1.33; *t*(9) = 1.52; *p* = 0.16).

#### Free Delayed Recall

A total of 334 patients with bvFTD and 912 patients with AD from 11 studies were included in the meta-analysis. The study by Teichmann et al. (2017) was omitted from the original pull of 12 records due to being identified as an outlier (see Supplementary Materials). The overall weighted effect size for bvFTD versus AD was 1.14 (95% CI [0.84, 1.45]), indicating that AD patients performed worse on Free Delayed Recall subtest compared to the bvFTD patients. Heterogeneity was tau = 0.39 (95% CI [0.16, 0.73]) (Fig. 3C). The Egger’s test did not reveal significant evidence of publication bias (bias: 2.00; *t*(9) = 1.67; *p* = 0.13).

#### Total Delayed Recall

A total of 349 patients with bvFTD and 871 patients with AD from 11 studies were included in the meta-analysis. The studies by Teichmann et al. (2017) and Cerciello et al. (2017) were omitted from the original pull of 13 records as they were identified as outliers (see Supplementary Materials for more details). The overall weighted effect size for bvFTD versus AD was 1.00 (95% CI [0.75, 1.28]), indicating that AD patients performed worse on the Total Delayed Recall subtest compared to the bvFTD patients. Heterogeneity was tau = 0.31 (95% CI [0.06, 0.65]) (Fig. 3D). Egger’s test was not significant (bias: 0.94; *t*(9) = 0.78; *p* = 0.45), so we have no evidence of publication bias.

### Qualitative Assessment

Two studies were deemed unsatisfactory, three studies were categorized as satisfactory, and eleven studies were classified as good studies (see Table 2). It is worth noting that the criterion regarding non-responders does not apply in the present context, impacting the validity of the total scores classification. The table provides more detailed information on specific subscores rather than overall scores. The absence of heterogeneity indicates the estimated effect size was not influenced by variations in study quality levels.

## Discussion

The relative preservation of memory is currently considered a diagnostic criterion for bvFTD, leading some to view amnesia as an exclusion criterion. In contrast, amnesia is a longstanding core clinical diagnostic feature of typical AD (Dubois et al., 2007). Consequently, neuropsychological assessment of memory impairments plays a pivotal role in the diagnosis of both bvFTD and AD, as well as in differentiating between these diseases. However, recent literature has shown that bvFTD might be exhibit long-term memory deficits, which in some cases may be as severe as those observed in AD patients (Hornberger & Piguet, 2012; Hornberger et al., 2010; Irish et al., 2014; Pennington et al., 2011). There is still ongoing debate regarding the nature of the memory difficulties experienced by patients with bvFTD. On the one hand, some authors suggested that bvFTD patients exhibit apparent amnesia, which primarily stems from deficits in strategic encoding and retrieval (Cerciello et al., 2017; Frisch et al., 2013; Glosser et al., 2002; Lemos et al., 2014; Pasquier et al., 2001; Pennington et al., 2011; Thomas-Anterion et al., 2000). On the other hand, other authors have reported cases of genuine amnesia that cannot be solely attributed to executive dysfunctions (Bertoux et al., 2014, 2016a; Cerami et al., 2016; Fernández-Matarrubia et al., 2017; Matuszewski et al., 2006). As a result, there is an ongoing debate concerning the presence of apparent versus genuine amnesia in bvFTD patients.

In the current study, we conducted a systematic review and meta-analysis of memory dysfunctions in patients with bvFTD, as measured by the FCSRT. This allowed us to identify the presence and severity of genuine amnesia and distinguish it from apparent amnesia.

In the meta-analysis, we included 16 studies that assessed memory deficits using the FCSRT. The samples included patients with a wide range of ages (57–75 years), education (6–15 years), and MMSE scores (14–29). These studies demonstrated that the FCSRT can be effectively administered to diverse subjects, making it a suitable instrument for memory assessment. Additionally, the test was administered in various languages (English, French, Spanish, Italian, and Portuguese), emphasizing the availability of normative*.* The meta-analysis results revealed significant differences in memory performance between patients with bvFTD and cognitively unimpaired participants (UP), indicating notable impairments in memory function among bvFTD patients. However, there were more similarities in memory performance between patients with bvFTD and those with AD.

A poor performance in the Total (Free + Cued) Recall scores (immediate and delayed) of the FCSRT, which reflects memory storage abilities, is considered a neuropsychological marker of genuine amnesia. Our findings demonstrated that, on average, bvFTD patients perform around one standard deviation worse than UP. The large effect size of this finding strongly suggests that bvFTD patients do suffer from a genuine form of amnesia. This result is additionally supported by statistical indicators of a lack of publication bias and a relatively small heterogeneity in the data. We also found that UP outperformed bvFTD on Free (Immediate and Delayed) Recall. The performance was about two standard deviations worse for the patients, which is an effect size twice as big as that of the total subscore, suggesting the presence of secondary memory deficits (i.e. encoding or retrieval difficulties).

The difference between UP and bvFTD in Total Delayed Recall (i.e. Pooled Effect Size of − 1.25) is much smaller than in Free Delayed Recall (i.e. Pooled Effect Size of − 2.19). Again, this suggests that bvFTD is characterized by both genuine and secondary memory deficits. Moreover, it is worth noting that bvFTD patients outperformed AD patients in all the FCSRT scores by almost one standard deviation (i.e. a strong effect size). This result indicates that memory difficulties observed in the bvFTD group are, on average, less severe than those in the AD group.

The present study indicates that bvFTD patients may exhibit genuine amnesia, which is evident compared to healthy individuals. At the same time, it is less severe than that observed in AD, placing bvFTD somewhat between the two groups. This result aligns with a previous meta-analysis that demonstrated bvFTD patients performing memory tests at an intermediate level between UP and AD patients, even if that study was unable to differentiate between genuine and secondary deficits (Poos et al., 2018). It is also consistent with the bimodal distribution retrieved by Bertoux et al. (2014), which suggested the presence of variability in memory performance within the bvFTD group, ranging from severe to subnormal FCSRT scores. This hypothesis is further supported by the variability observed in the Total Immediate Recall scores within the samples included in our meta-analysis (average standard deviation: AD = 8.87; bvFTD = 9.84; UP = 3.11). The higher variability in bvFTD patients aligns with the expected pattern in cases of bimodal distributions. While Poos et al. (2018) confirmed the presence of memory impairment in bvFTD, our study was able to distinguish between the contributions of genuine and apparent amnesia and determine the actual level of deficit in bvFTD as compared to AD and UP, which had not been done previously.

Overall, the present results suggest the coexistence of that both genuine and apparent amnesia in bvFTD and AD patients. We found strong evidence of genuine amnesia, while also observing clear indications that secondary deficits worsen the performance of bvFTD patients. The group effect could be attributed to a bimodal performance distribution (Bertoux et al., 2014) or it may be indicative of a generally distributed decreased performance across the entire population. This question cannot be answered with a group-level meta-analytic approach, and we warn from simplistic interpretations of the results at the individual level.

Although in research studies, bvFTD patients can be differentiated from AD patients at the group level based on their memory performance, this distinction does not hold true at the individual—and, therefore, clinical—level (Bertoux et al., 2018; Frisch et al., 2013; Hutchinson & Mathias, 2007; Mansoor et al., 2015; Poos et al., 2018). Even when using the most discriminating memory measurements, such as the FCSRT, for individual-level assessment, the clinical differential diagnosis between AD and bvFTD remains challenging, solely relying on memory performance (Hutchinson & Mathias, 2007; Poos et al., 2018). According to Zakzani’s calculations of overlap statistics (Zakzani, 2001), the estimated effect size indicates a percentage of overlap between AD and bvFTD of approximately 40%, whether considering the total or the free recall. This further highlights the challenge of distinguishing between AD and bvFTD at the individual level using memory tests alone. Recently, it has been suggested to differentiate between two subtypes of bvFTD: amnesic-bvFTD and non-amnesic-bvFTD. Approximately 50% of bvFTD patients fall into the amnesic-bvFTD category, and they exhibit severe impairment in both FCSRT and “conventional” memory tests based on free recall. Furthermore, these patients also show alterations in medial temporal structures (Bertoux et al., 2014, 2016a, b; Cerami et al., 2016; Fernández-Matarrubia et al., 2017; Ramanan et al., 2017). As emphasized previously, the dual profile of bvFTD patients, with both amnestic and non-amnestic presentation, explains why average memory scores can be statistically different between AD and bvFTD at a group level but not at the individual level (Bertoux et al., 2018).

From a clinical perspective, our study suggests that any future consensual revision of the current diagnostic criteria for bvFTD should no longer include the relative preservation of episodic memory as a neuropsychological subcriterion. This approach has already been adopted in the recently proposed diagnosis criteria for prodromal bvFTD by the ALLFTD group (Barker et al., 2022), considering the limited specificity of memory disorders in distinguishing bvFTD from other neurodegenerative diseases, despite their reasonable sensitivity. However, further studies are needed to confirm this result. In the last diagnostic criteria of FTD (Neary et al., 1998), and subsequently for bvFTD (Rascovsky et al., 2011), significant emphasis was placed on episodic memory as it was believed to enable effective differentiation from AD, which is the most common differential diagnosis for bvFTD (Hornberger & Piguet, 2012; Bertoux et al., 2018). However, given the high proportion of amnesic-bvFTD patients, the limited sensitivity of memory assessment to Alzheimer’s pathology (e.g. Bertoux et al., 2020), and the revised criteria of Alzheimer’s disease based on biological markers (Jack et al., 2018), such a focus is no longer relevant. Instead, other neuropsychological domains or tests (i.e. navigation, praxis, or social cognitive abilities) may be more promising and should be given preference if they demonstrate high discriminative power between bvFTD and AD or primary psychiatric disorders, which are often considered the second differential diagnosis (Bertoux et al., 2016b; Yew et al., 2013). Additionally, in our study, the effect size of the AD vs bvFTD comparison was equivalent for both the total recall and the free recall, suggesting that the two groups exhibit a similar performance pattern, albeit at different levels of severity. In other words, apparent amnesia appears to impact the performance of AD patients as well as it does in bvFTD patients. The clinical differential diagnosis between bvFTD and AD is challenging, and although the FCSRT is a sensitive and helpful test, additional information is essential for an accurate diagnosis. While the differentiation between genuine and secondary amnesia is relevant, as it is based on the associated cerebral damage, the results of the present meta-analysis demonstrate the presence of both deficits in various types of cognitive impairment. This finding aligns with the understanding that memory mechanisms work in conjunction for efficient functioning. When clinicians encounter memory loss, it is likely that multiple cognitive mechanisms are involved to varying degrees. Therefore, it may be advantageous to reevaluate the tendency to consider each function in isolation and instead adopt a more comprehensive perspective on cognitive functioning (Ferguson, & Alzheimer’s Disease Neuroimaging Initiative, 2021; Tosi et al., 2020).

Our study highlights a genuine impairment of long-term memory in bvFTD through a rigorous meta-analysis. Consequently, it presents a compelling case to revise the neuropsychological criteria of bvFTD and the current clinical practices. Our meta-analysis employed modern and rigorous indicators and followed the latest international guidelines, which—we believe—strengthened our findings. The use of Bayesian modelling and the availability of our data to the community can be viewed as the initial phase of an incremental collaborative study that holds the potential to refine or corroborate our results through the inclusion of future research.

However, it is important to acknowledge some limitations of our study. Firstly, as is customary in the field, we considered Rascovsky et al., 2011 (or Neary et al., 1998) criteria as a condition for studies to be included in our meta-analysis. However, strictly applying these criteria would have resulted in the exclusion of severely amnestic patients from the original studies. Consequently, the proportion of amnestic bvFTD patients is likely higher than what is reported, potentially leading to an underestimation of the estimated effect size in our study. Additionally, bvFTD is already known to be underdiagnosed (and memory impairment likely contributes to this underdiagnosis). Therefore, our estimated effect size can be viewed as a lower limit of the possible effect, with the actual effect size likely being higher.

Furthermore, dementia syndromes such as AD and bvFTD can involve “mixed” neuropathological processes, which can only be definitively distinguished through autopsy confirmation. Unfortunately, we identified only one study in our meta-analysis that reported neuropathologically proven cases of bvFTD. As a result, we were unable to conduct further analysis specifically focused on autopsy-proven cases. In the present meta-analysis, we are unable to differentiate memory impairment as a function of bvFTD or mixed bvFTD with other neurodegenerative syndromes (or its potential co-occurrence with other neurodegenerative syndromes). The studies we reviewed mostly reported clinical diagnoses, without accounting for pathology data or co-morbid pathology. Therefore, our results should be considered with a degree of imprecision due to the general lack of pathologically proven diagnoses. However, evidences ranging from pathological cases to group-studies have reported memory deficit associated with confirmed FTD (see for example Hornberger & Piguet, 2012; Hornberger et al., 2012; Bertoux et al., 2020). Notably, the present meta-analysis included one study with definite diagnoses (Bertoux et al., 2020), which compared bvFTD patients with FTLD pathology to confirmed AD patients. The effect size found by the authors lay near the lower bound of the pooled effect credible intervals, indicating a possible slightly smaller difference between AD and bvFTD compared to other studies in the meta-analysis. Nonetheless, this study did not emerge as an influential point or an outlier in our preliminary analyses, providing no evidence to consider it differently from those studies relying solely on clinical diagnoses. Further research involving definitive diagnosis would be beneficial in drawing more conclusive findings regarding the amnestic profile of bvFTD. However, studies conducted using the FCSRT in genetic FTD populations have reported a similar pattern to the one we observed in our analysis. While not all patients in those studies received a bvFTD diagnosis (although it was the most frequent presentation), both apparent and genuine memory deficits are recognized as integral components of the clinical spectrum in genetic FTD (Poos et al., 2021, 2022; Tavares et al., 2020).

Another limitation of our study is that, due to our rigorous methodology and stringent inclusion criteria, we were only able to include 16 studies from a large pool of available research. While the inclusion of studies may have its drawbacks, the strength of this research lies in the high quality of the included studies. This is evident from the quality assessment table, which demonstrates the absence of publication bias and heterogeneity, thus enhancing the reliability of our results. In addition, it is important to note that out of the 16 papers included, four studies (Alcolea et al., 2019; Cercielloet al., 2017; Canu et al., 2017; Teichman et al., 2017) exhibited extreme effect sizes and were consequently excluded from the analyses as outliers. Alcolea et al. (2019) and Cerciello et al. (2017) used a 24-item version of the Grober-Buschke test instead of the classical 16-item version. Furthermore, both Alcolea et al. (2019) and Teichmann et al. (2017) had larger sample sizes compared to the other studies included in the meta-analysis. On the one hand, the usage of different test versions may have resulted in varied impacts on the subjects’ performance, while the imbalanced sample sizes have influenced the calculation of effect sizes. Additionally, Canu et al. (2017) reported more severe impairment in the AD patients compared to the bvFTD group, based on the CDR score. This discrepancy may have contributed to poorer FCSRT performances in the AD patients group, relative to the other studies included in the meta-analysis.

Another limitation of this meta-analysis was its focus on a single memory test. We selected the FCSRT as it is widely recommended for assessing verbal memory in dementia, as indicated Table S1 in Boccardi et al. (2021). The FCSRT also offers the clinical advantage of controlling for effective encoding through a “search procedure”, followed by repeated free and cued recall trials. Another prominent memory test, the California Verbal Learning Test (CVLT), shares a similar feature, incorporating a cued recall phase but not controlling the encoding phase. We aimed to include the CVLT in our meta-analysis to draw more comprehensive conclusions. Unfortunately, the published studies did not report all the necessary information, and the authors we contacted were unable to supply sufficient information to construct a CVLT database. We have documented the results of the systematic review search in the Supplementary Materials.

In conclusion, our study demonstrates that bvFTD is characterized by genuine amnesia as assessed by the FCSRT. The deficit is substantial and falls between the performance of healthy individuals and that of AD patients’ performance, highlighting its clinical relevance for both the diagnosis of bvFTD and the differentiation between bvFTD and AD based on the FCSRT. Importantly, bvFTD patients exhibit evident signs of amnesia. Our meta-analysis confirms the presence of a memory deficit in bvFTD patients, supporting the coexistence of both genuine and secondary memory deficits. However, our findings also raise new questions. For instance, what is the interplay between primary and secondary deficits? Do they emerge simultaneously? Future meta-analytic studies should explore whether bvFTD patients present less severe deficits or exhibit diverse profiles that may include or exclude memory impairment, such as a potential bimodal distribution within the bvFTD population. As memory assessment is insufficient for an accurate and reliable clinical differentiation between bvFTD and AD, further investigation is needed to establish the best strategy for the differential diagnosis. For instance, future meta-analyses could incorporate additional neuropsychological measures to enhance the characterization of bvFTD characterization. Unravelling the pathway of genuine and secondary deficit in patients with bvFTD (and AD) is a crucial step toward improving our understanding of these conditions, with potential implications for early and late diagnosis. We believe that our study represents an important contribution in that direction.

## Supplementary Information

Below is the link to the electronic supplementary material.

Supplementary file1 (DOCX 7413 KB)

## Data Availability

Data and analysis scripts are available on the OSF platform at the following link: https://osf.io/eazck/?view_only=790c6423122a4583960bc9190167924b.
