# Supplementary material for: Genuine Memory Deficits as Assessed by the Free and Cued Selective Reminding Test (FCSRT) in the Behavioural Variant of Frontotemporal Dementia. A Systematic Review and Meta-analysis Study
Source: Neuropsychol Rev. 2023 Sep 22;34(3):823–37. doi: 10.1007/s11065-023-09613-3 (PMC11473568; doi:10.1007/s11065-023-09613-3)
Supplement: Supplementary file 1 — Supplementary file1 (DOCX 7413 KB) [file 11065_2023_9613_MOESM1_ESM.docx]

**Supplementary Materials**

This supplementary material file contains the modified version Newcastle-Ottawa rating scale and a few additional analyses aimed at full transparency in data analysis and reports. Specifically:

1. we report analysis aimed at identifying outliers in input data;
2. we report additional information about the model convergence presented in the main text;
3. we report a prior robustness check to verify the prior selection's impact on the results;
4. we report the search outcome of a systematic review focused on the California Verbal Learning Test.
5. **Newcastle-Ottawa rating scale (adapted for cross-sectional studies).**

| Item | Star to assign if the paper meets the requirement | Requirement |
| --- | --- | --- |
| **Selection: (Maximum 5 stars)** | | |
| 1. Representativeness of the sample: | | |
| - 1. Truly representative of the average in the target population (all subjects or random sampling). | 1 | Multicentric studies. |
| - 1. Somewhat representative of the average in the target population (non-random sampling). | 1 | Patients consecutively recruited in a Hospital. |
| - 1. Selected group of users. | 0 | Subjects with specific characteristics (not of interest).  E.g., women or university students |
| - 1. No description of the sampling strategy. | 0 | No description of the sampling strategy. |
| 1. Sample size: |  |  |
| - 1. Justified and satisfactory. | 1 | The sample size of each group is justified and satisfactory.  E.g., the a-priori power analysis is reported |
| - 1. Not justified. | 0 | No justification of the sample size |
| 1. Non-respondents: |  |  |
| - 1. Comparability between respondents and non-respondents characteristics is established, and the response rate is satisfactory. | 1 | NA (this point does not apply to the current situation) |
| - 1. The response rate is unsatisfactory, or the comparability between respondents and non-respondents is unsatisfactory. | 0 |  |
| - 1. No description of the response rate or the characteristics of the responders and the non-responders. | 0 |  |
| 1. Ascertainment of the exposure (risk factor): | | |
| - 1. Validated measurement tool. | 2 | Diagnosis is made following diagnostic criteria |
| - 1. Non-validated measurement tool, but the tool is available or described. | 1 | Diagnosis is not based on diagnostic criteria, but the authors explain the rationale |
| - 1. No description of the measurement tool. | 0 | The description of the diagnostic procedure is not reported |
| **Comparability: (Maximum 2 stars)** | | |
| 1. The subjects in different outcome groups are comparable based on the study design or analysis. Confounding factors are controlled. | | |
| - 1. The study controls for the most important factor (select one). | 1 | The study controls for differences in impairment severity between bvFTD and AD through the MMSE and/or the CDR.  Specify:  MATCHED if the groups are matched by severity (i.e., there are no significant differences)  CONTROLLED if the severity differs between the group, but this is controlled in the analyses (i.e., severity is used as a covariate) |
| - 1. The study control for any additional factor. | 1 | The study controls for differences in age, school age, or sex between bvFTD and AD.  Specify:  MATCHED if the groups are matched (i.e., there are no significant differences)  CONTROLLED if there is a significant difference between the group, but this is controlled in the analyses (i.e., the variable is used as a covariate)  Specify the contrasts (bvFTD vs HC; bvFTD vs AD; AD vs HC) |
| **Outcome: (Maximum 3 stars)** | | |
| 1. Assessment of the outcome: | | |
| - 1. Independent blind assessment. | 2 | An external neuropsychologist administered the FCSRT (blinded). |
| - 1. Record linkage. | 2 | Data are derived from a database, and the neuropsychological evaluation was not administered by one of the authors of the manuscript (blinded). |
| - 1. Self-report. | 1 | The neuropsychological evaluation was administered by one of the authors of the manuscript (open-label). |
| - 1. No description. | 0 | It is not specified who administered the neuropsychological evaluation |
| 1. Statistical test: | | |
| - 1. The statistical test used to analyze the data is clearly described and appropriate, and the measurement of the association is presented, including confidence intervals and the probability level (p-value). | 1 | The statistical test used to analyze the FCSRT is clearly described (when it is the focus of the paper), and confidence intervals and/or the probability level (p-value) are reported. |
| - 1. The statistical test is not appropriate, not described, or incomplete. | 0 | One of the following cases:  The statistical test is not described  The statistical test is not appropriate  The analyses results are incomplete or not reported |

1. **Outliers identification**

We used the R function "find.outliers" in the package "dmetar" to check for the presence of outliers ). Here follows the original pull of records before the outliers removal.

The following tables report the Standardized Mean Difference (SMD); its related upper and lower bound of the confidence interval (95% CI); the weight of each paper. The weight is calculated as a ratio of sample size over precision, and here it is expressed as a percentage of the cumulated weights (%Weight). An asterisk highlights the papers identified as outliers in the homonymous column.

- 1. *bvFTD vs cognitively unimpaired participants*

*Total Immediate Recall*

| *Study* | *SMD* | *95%CI Lower* | *95%CI Upper* | *%Weight* | *Outlier* |
| --- | --- | --- | --- | --- | --- |
| *Lage, 2020* | -1,42 | -2,08 | -0,76 | 9,71 |  |
| *Matuszewski, 2006* | -1,43 | -2,13 | -0,74 | 9,23 |  |
| *Piolino, 2003* | -1,44 | -2,21 | -0,66 | 8,14 |  |
| *Gala, 2019* | -2,19 | -2,47 | -1,92 | 16,42 | * |
| *Lemos, 2014* | -1,32 | -1,86 | -0,78 | 11,55 |  |
| *Bertoux, 2018* | -1,01 | -1,67 | -0,35 | 9,69 |  |
| *Fernandez-Matarrubia, 2017* | -1,30 | -1,91 | -0,68 | 10,39 |  |
| *Bertoux, 2014* | -1,15 | -1,70 | -0,60 | 11,44 |  |
| *Bertoux, Ramanan, 2016* | -1,21 | -1,65 | -0,77 | 13,43 |  |

*Total Delayed Recall*

| Study | SMD | *95%CI Lower* | *95%CI Upper* | *%Weigth* | *Outlier* |
| --- | --- | --- | --- | --- | --- |
| *Lage, 2020* | -1,74 | -2,44 | -1,05 | 9,32 |  |
| *Matuszewski, 2006* | -1,08 | -1,74 | -0,42 | 9,66 |  |
| *Piolino, 2003* | -1,33 | -2,09 | -0,56 | 8,63 |  |
| *Gala, 2019* | -2,20 | -2,47 | -1,92 | 13,49 | * |
| *Cerciello, 2017* | -3,00 | -4,14 | -1,85 | 5,71 | * |
| *Lemos, 2014* | -1,70 | -2,27 | -1,12 | 10,52 |  |
| *Bertoux, 2018* | -1,16 | -1,84 | -0,49 | 9,52 |  |
| *Fernandez-Matarrubia, 2017* | -1,24 | -1,85 | -0,63 | 10,17 |  |
| *Bertoux, 2014* | -0,81 | -1,34 | -0,28 | 10,99 |  |
| *Bertoux, Ramanan, 2016* | -1,12 | -1,55 | -0,68 | 12,01 |  |

- 1. *bvFTD vs AD*

*Free Immediate Recall*

| *Study* | *SMD* | *95%Ci Lower* | *95%Ci Upper* | *%Weigth* | *Outlier* |
| --- | --- | --- | --- | --- | --- |
| *Lage, 2020* | 1,35 | 0,61 | 2,09 | 9,04 |  |
| *Bertoux, 2020* | 0,49 | -0,11 | 1,08 | 9,09 |  |
| *Pozueta, 2019* | 0,77 | 0,19 | 1,35 | 9,10 |  |
| *Canu, 2017* | 2,00 | 1,46 | 2,54 | 9,11 | * |
| *Boutoleau-Bretonnière, 2015* | 0,41 | -0,13 | 0,95 | 9,11 |  |
| *Gala, 2019* | 0,99 | 0,77 | 1,21 | 9,17 |  |
| *Lemos, 2014* | 1,76 | 1,18 | 2,34 | 9,10 |  |
| *Teichmann, 2017* | 11,06 | 10,08 | 12,04 | 8,94 | * |
| *Bertoux, 2018* | 1,29 | 0,62 | 1,95 | 9,07 |  |
| *Bertoux, 2014* | 0,87 | 0,46 | 1,29 | 9,14 |  |
| *Basely, 2013* | 0,80 | 0,34 | 1,26 | 9,13 |  |

*Total Immediate Recall*

| *Study* | *SMD* | *95%Ci Lower* | *95%Ci Upper* | *%Weigth* | *Outlier* |
| --- | --- | --- | --- | --- | --- |
| *Lage, 2020* | 1,65 | 0,88 | 2,41 | 8,25 |  |
| *Bertoux, 2020* | 0,35 | -0,24 | 0,93 | 8,34 |  |
| *Pozueta, 2019* | 1,30 | 0,68 | 1,92 | 8,33 |  |
| *Boutoleau-Bretonnière, 2015* | 1,23 | 0,65 | 1,81 | 8,35 |  |
| *Piolino, 2003* | 1,17 | 0,36 | 1,98 | 8,23 |  |
| *Gala, 2019* | 0,90 | 0,67 | 1,12 | 8,46 |  |
| *Lemos, 2014* | 1,41 | 0,86 | 1,96 | 8,36 |  |
| *Teichmann, 2017* | 9,44 | 8,59 | 10,29 | 8,21 | * |
| *Bertoux, 2018* | 1,11 | 0,46 | 1,76 | 8,32 |  |
| *Fernandez-Matarrubia, 2017* | 1,32 | 0,73 | 1,91 | 8,34 |  |
| *Bertoux, 2014* | 0,93 | 0,52 | 1,35 | 8,41 |  |
| *Basely, 2013* | 0,71 | 0,25 | 1,16 | 8,40 |  |

*Free Delayed Recall*

| *Study* | *SMD* | *95%Ci Lower* | *95%Ci Upper* | *%Weigth* | *Outiler* |
| --- | --- | --- | --- | --- | --- |
| *Lage, 2020* | 1,43 | 0,68 | 2,18 | 8,28 |  |
| *Bertoux, 2020* | 0,42 | -0,18 | 1,01 | 8,35 |  |
| *Pozueta, 2019* | 0,44 | -0,13 | 1,00 | 8,36 |  |
| *Canu, 2017* | 1,91 | 1,37 | 2,44 | 8,37 |  |
| *Gala, 2019* | 0,79 | 0,57 | 1,02 | 8,45 |  |
| *Cerciello, 2017* | 1,79 | 0,80 | 2,79 | 8,15 |  |
| *Lemos, 2014* | 1,48 | 0,92 | 2,03 | 8,36 |  |
| *Teichmann, 2017* | 10,20 | 9,29 | 11,11 | 8,20 | * |
| *Bertoux, 2018* | 1,21 | 0,55 | 1,86 | 8,32 |  |
| *Fernandez-Matarrubia, 2017* | 1,26 | 0,68 | 1,85 | 8,35 |  |
| *Bertoux, 2014* | 1,20 | 0,77 | 1,63 | 8,40 |  |
| *Basely, 2013* | 1,11 | 0,64 | 1,58 | 8,39 |  |

*Total Delayed Recall*

| *Study* | *SMD* | *95%Ci Lower* | *95%Ci Upper* | *%Weigth* | *Outlier* |
| --- | --- | --- | --- | --- | --- |
| *Lage, 2020* | 1,78 | 1,00 | 2,57 | 7,60 |  |
| *Bertoux, 2020* | 0,31 | -0,27 | 0,90 | 7,74 |  |
| *Pozueta, 2019* | 0,81 | 0,22 | 1,39 | 7,74 |  |
| *Boutoleau-Bretonnière, 2015* | 0,56 | 0,02 | 1,11 | 7,77 |  |
| *Piolino, 2003* | 0,54 | -0,22 | 1,29 | 7,62 |  |
| *Gala, 2019* | 0,82 | 0,60 | 1,04 | 7,89 |  |
| *Cerciello, 2017* | 2,99 | 1,75 | 4,23 | 7,17 | * |
| *Lemos, 2014* | 1,47 | 0,91 | 2,02 | 7,76 |  |
| *Teichmann, 2017* | 7,94 | 7,21 | 8,67 | 7,64 | * |
| *Bertoux, 2018* | 1,22 | 0,57 | 1,88 | 7,69 |  |
| *Fernandez-Matarrubia, 2017* | 1,01 | 0,44 | 1,57 | 7,75 |  |
| *Bertoux, 2014* | 1,15 | 0,72 | 1,57 | 7,82 |  |
| *Basely, 2013* | 1,51 | 1,02 | 2,00 | 7,79 |  |

1. **Model Convergence check**

To assess the model convergence and the overall validity, we conducted posterior predictive checks, and we inspected the trace plots and the density plots for posterior samples. In the posterior predictive checks, data are simulated through random draws from the posterior distribution and then compared to the observed data. If a model has converged and captures the data well, the densities of the replications are roughly similar to the one of the observed data. As for the density plot, increasing amounts of data leads to a posterior distribution approaching multivariate normality. Moreover, we look for a trace plot that shows random scatter around a mean value, and where the chains mixed with one another and converge. A model that converges typically presents overlapping chains in the trace plots.

Here we reported, for each comparison, the posterior check, the density plot and the trace plot of the analysis presented in the main text.

The model started from a non-informative prior with a Normal distribution with a mean of 0 and a standard deviation of 10 for the effect size and a half-Cauchy distribution with a mean of 0 and a scale of .5 for the heterogeneity.

All the analyses showed a satisfactory convergence.

- 1. *bvFTD vs cognitively unimpaired participants*

*Free Immediate Recall*


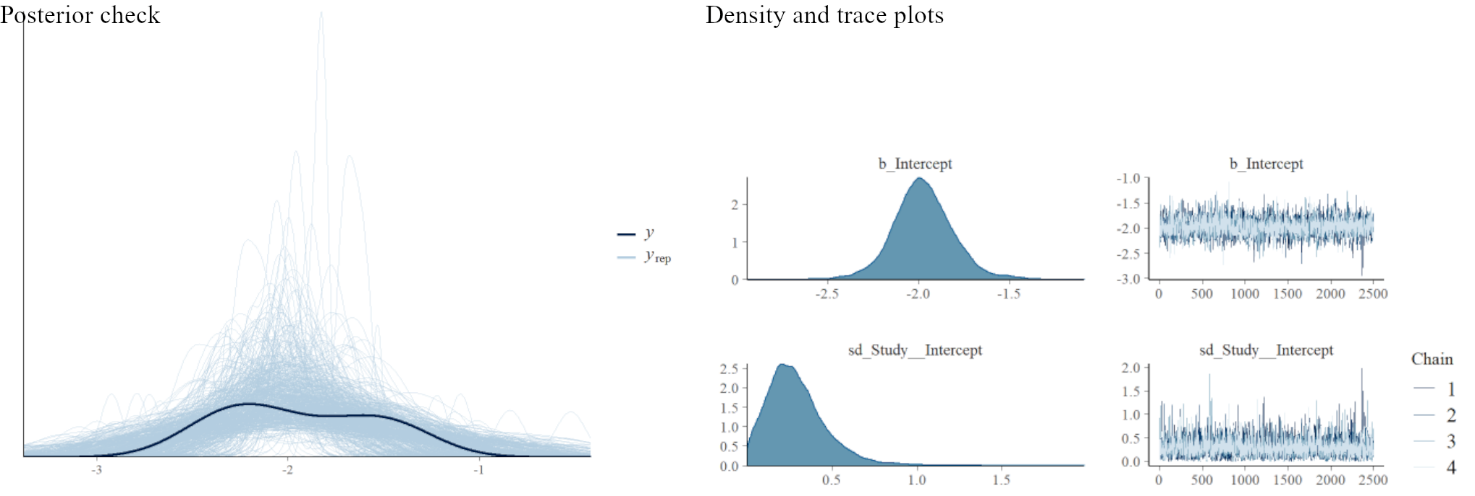


*Total Immediate Recall*


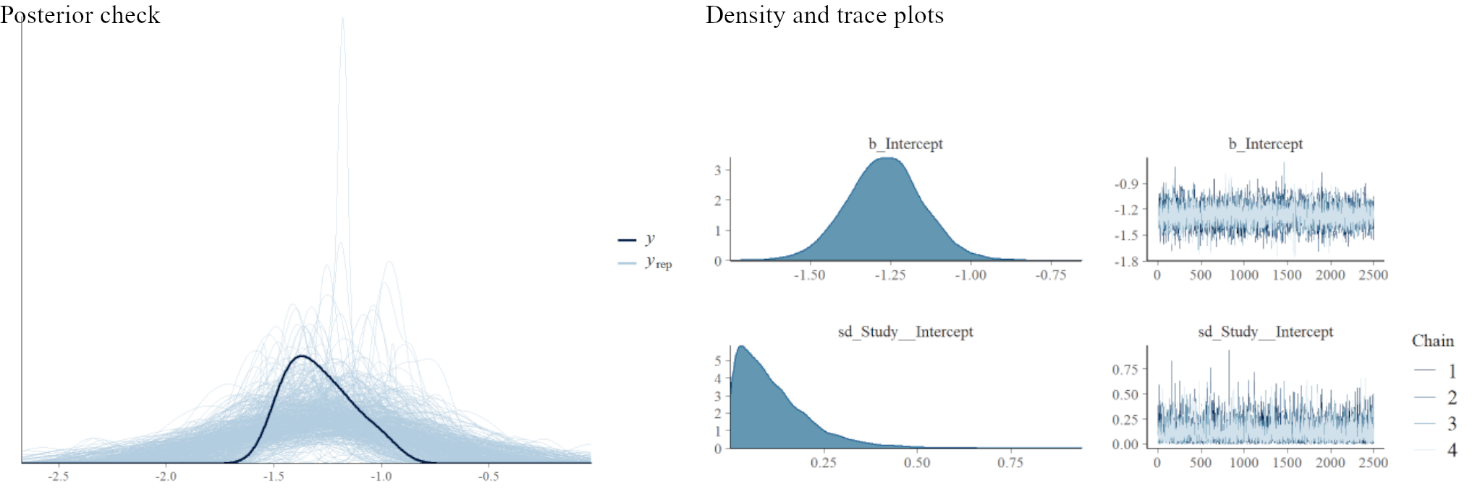


*Free Delayed Recall*


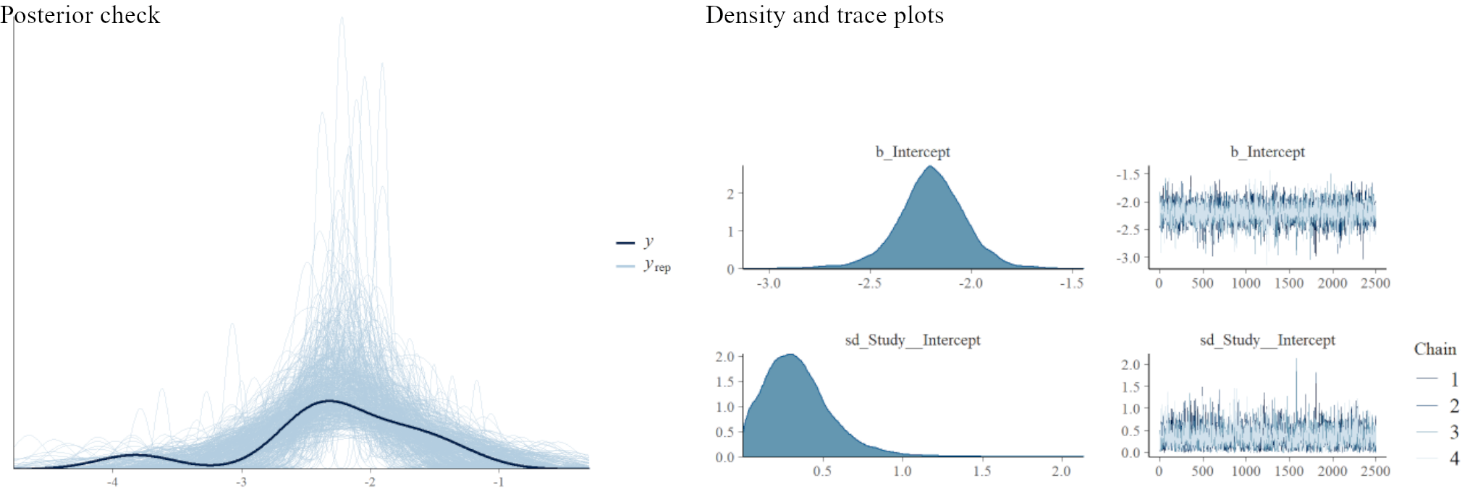


*Total Delayed Recall*


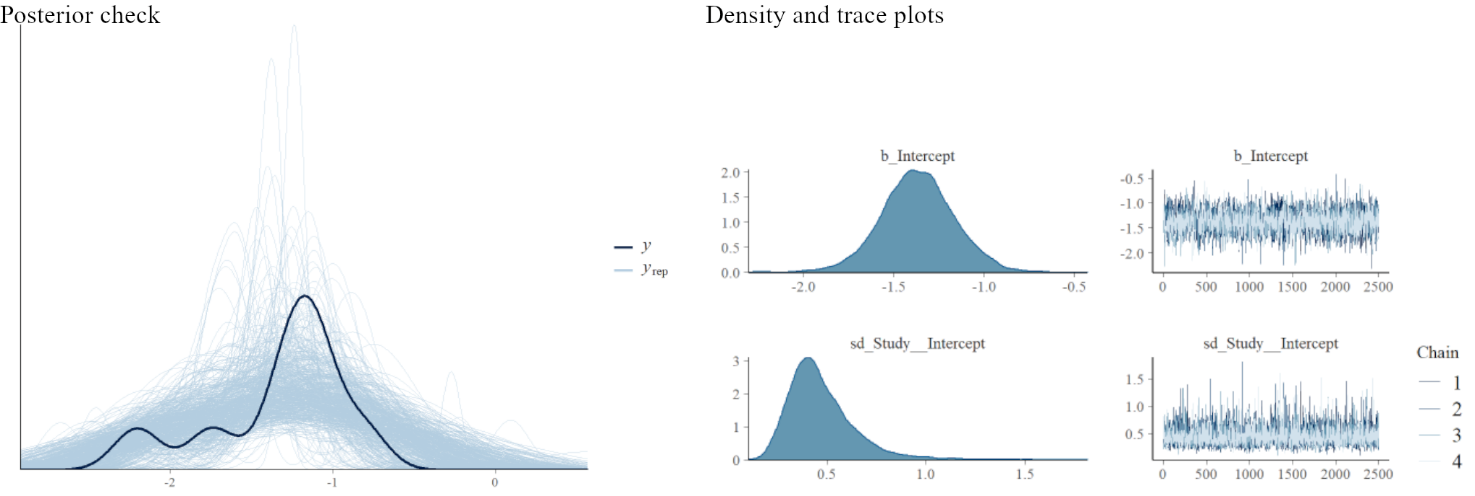


- 1. *bvFTD vs AD*

*Free Immediate Recall*


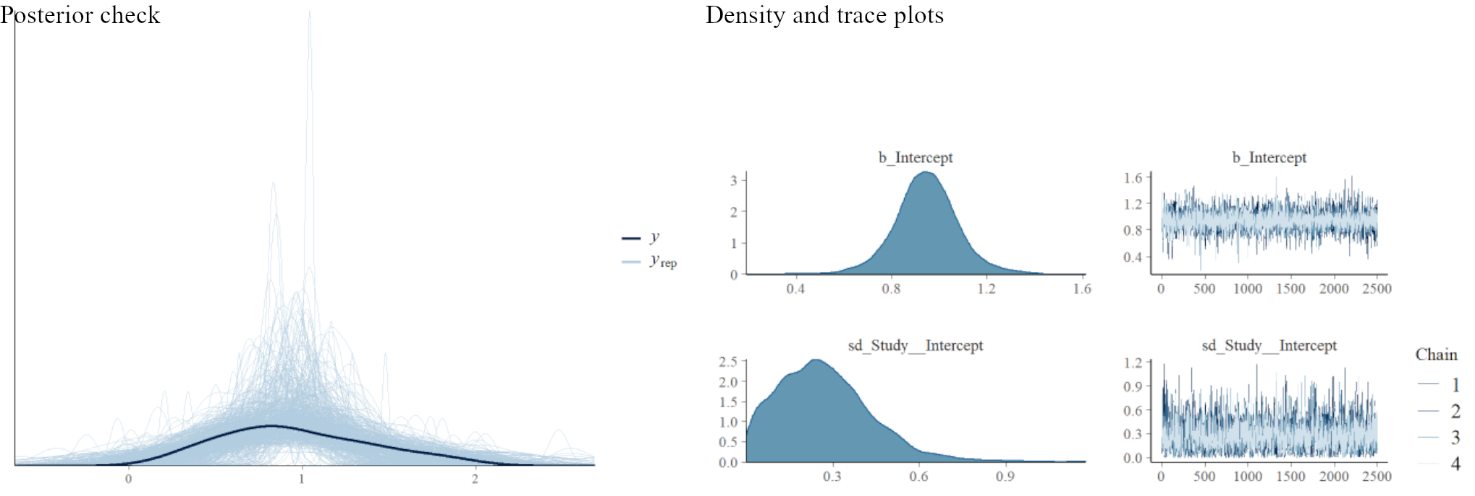


*Total Immediate Recall*

*
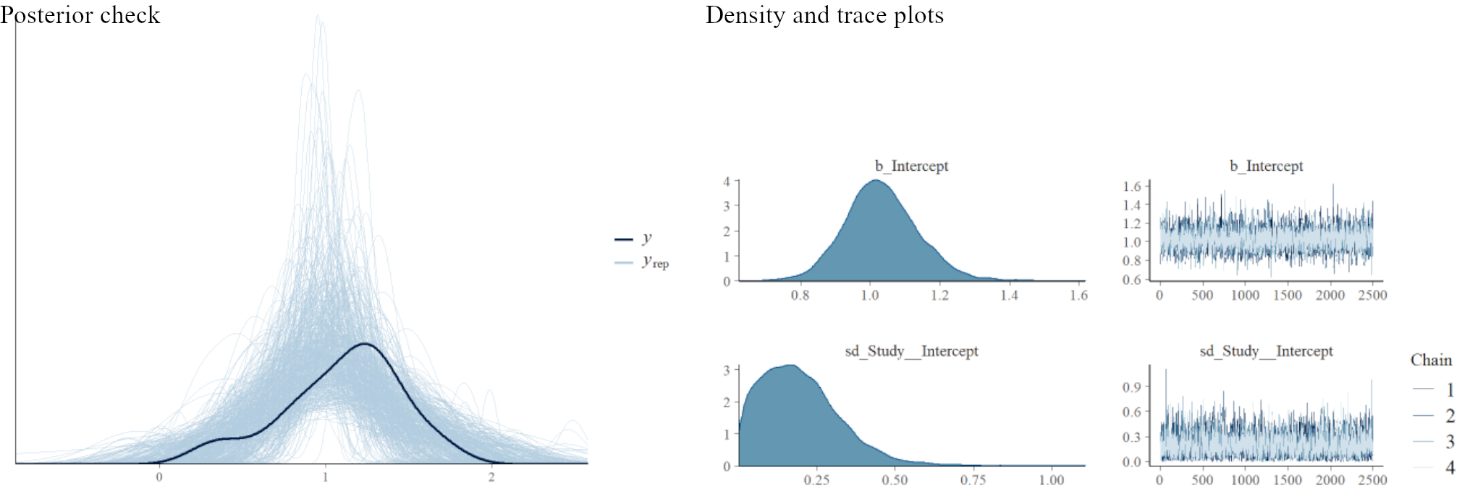
*

*Free Delayed Recall*

*
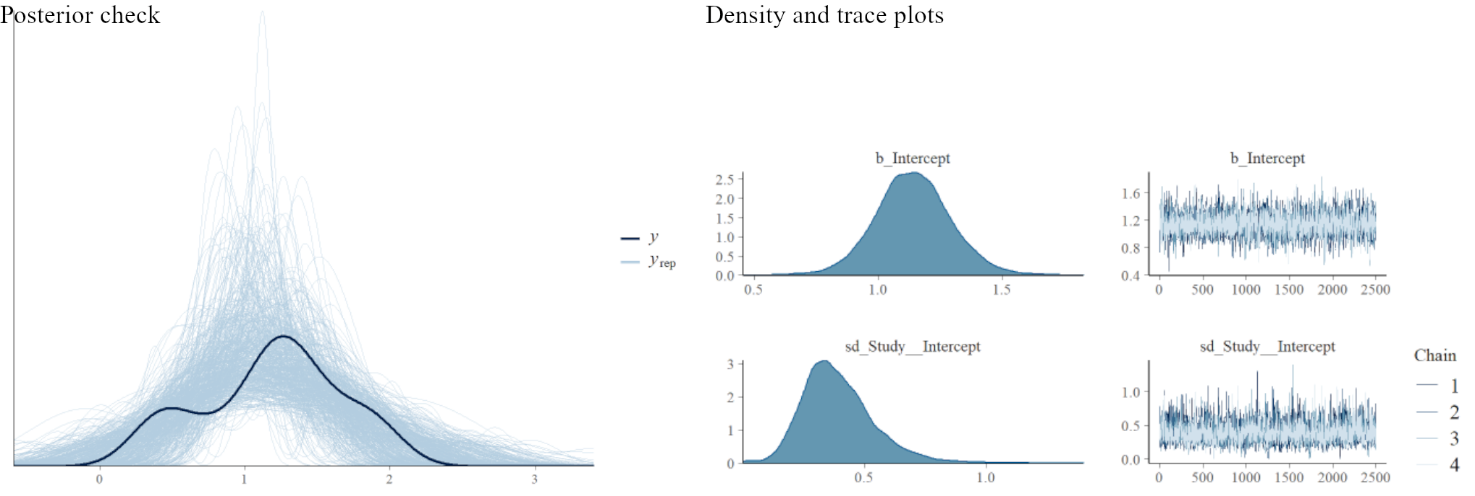
*

*Total Delayed Recall*


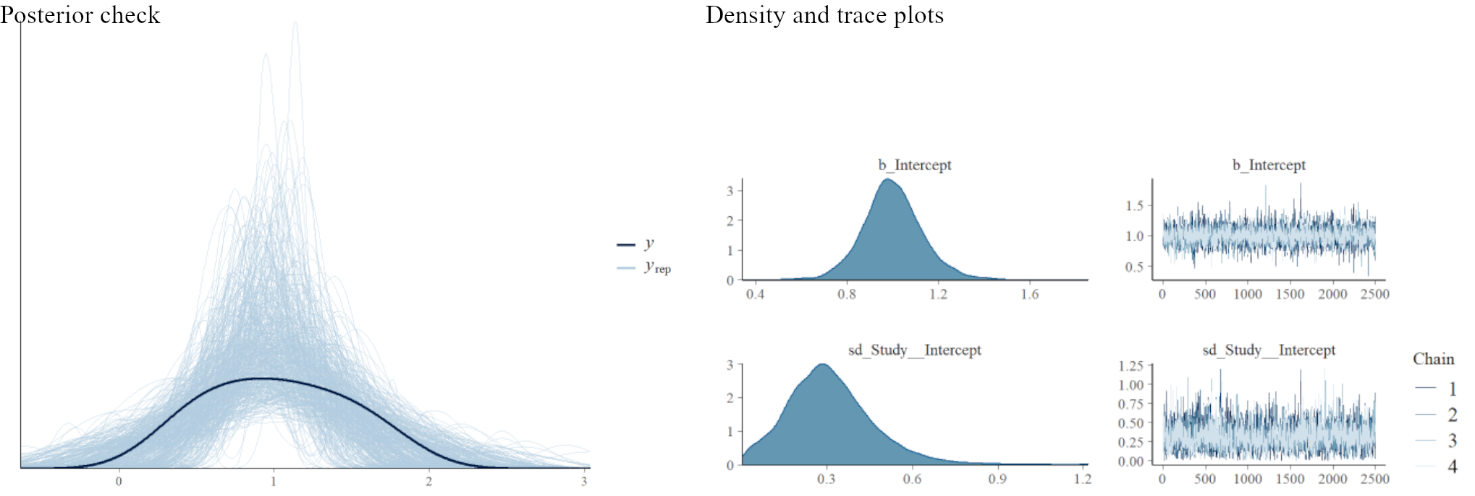


1. **Prior robustness check**

We also provide the model convergence and the forest plot for two alternative prior distributions of the effect size to verify the robustness of the results. As alternative priors we employed:

1. a wide prior using a Normal distribution with mean 0 and a scale of 5
2. an informative prior using a Normal distribution with mean 0 and a scale of 1.

We did not change the prior distribution for the heterogeneity.

Overall, the pooled effect sizes resulted to be stable when we used different prior distributions. The scaling parameter controls how much the distribution spreads out to lower and higher values as respect to the mean of the distribution. In other words, the distribution scale controls how much we are confident that the true effect will be close to the prior distribution mean. The lower the scale, the more informative the prior distribution will be. In the computation of the posterior distribution, informative priors have a higher influence than non-informative or weakly-informative priors.

In the main text, we started with a non-informative prior (mean = 0; scale = 10) that is likely to have a very low impact on the computation of the pooled effect size. Here, we checked for the stability of the results by trying more informative prior distributions. The results did not change much, suggesting that the pooled effect sizes is only marginally impacted by the subjective component of the Bayesian analyses and thus we can conclude that results are stable and reliable.

- 1. *bvFTD vs cognitively unimpaired participants*

*Free Immediate Recall*

a)
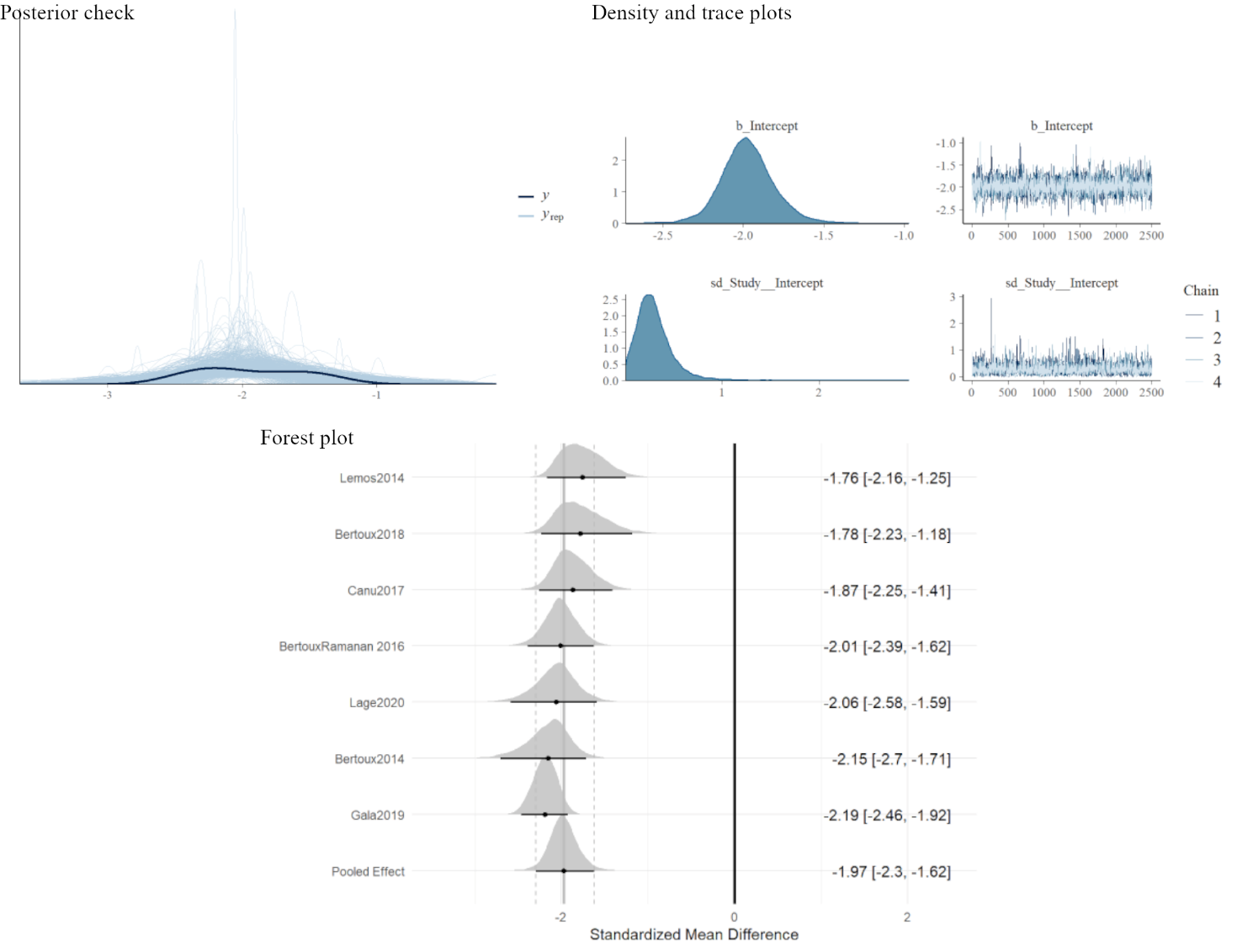


b)
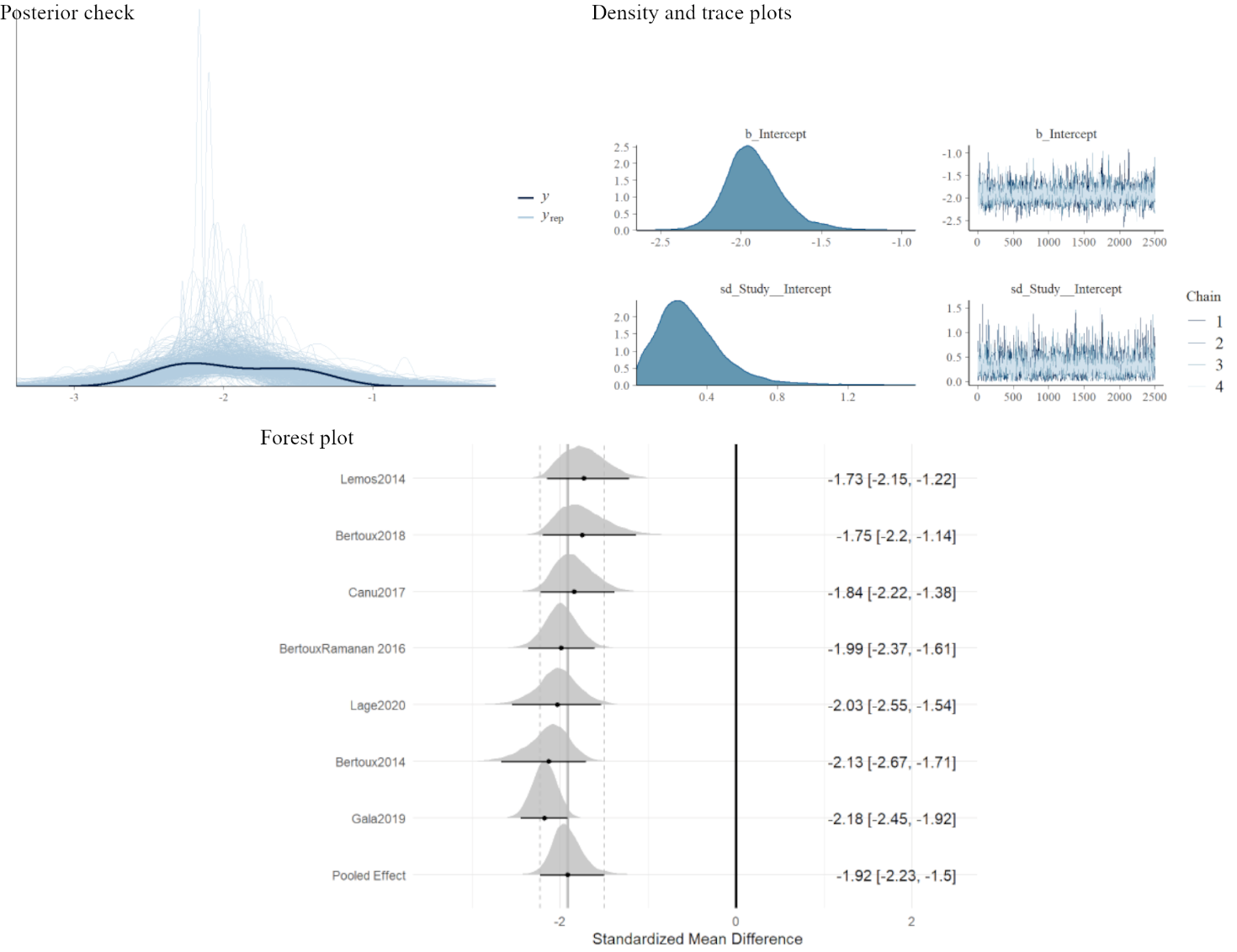


*Total Immediate Recall*

a)
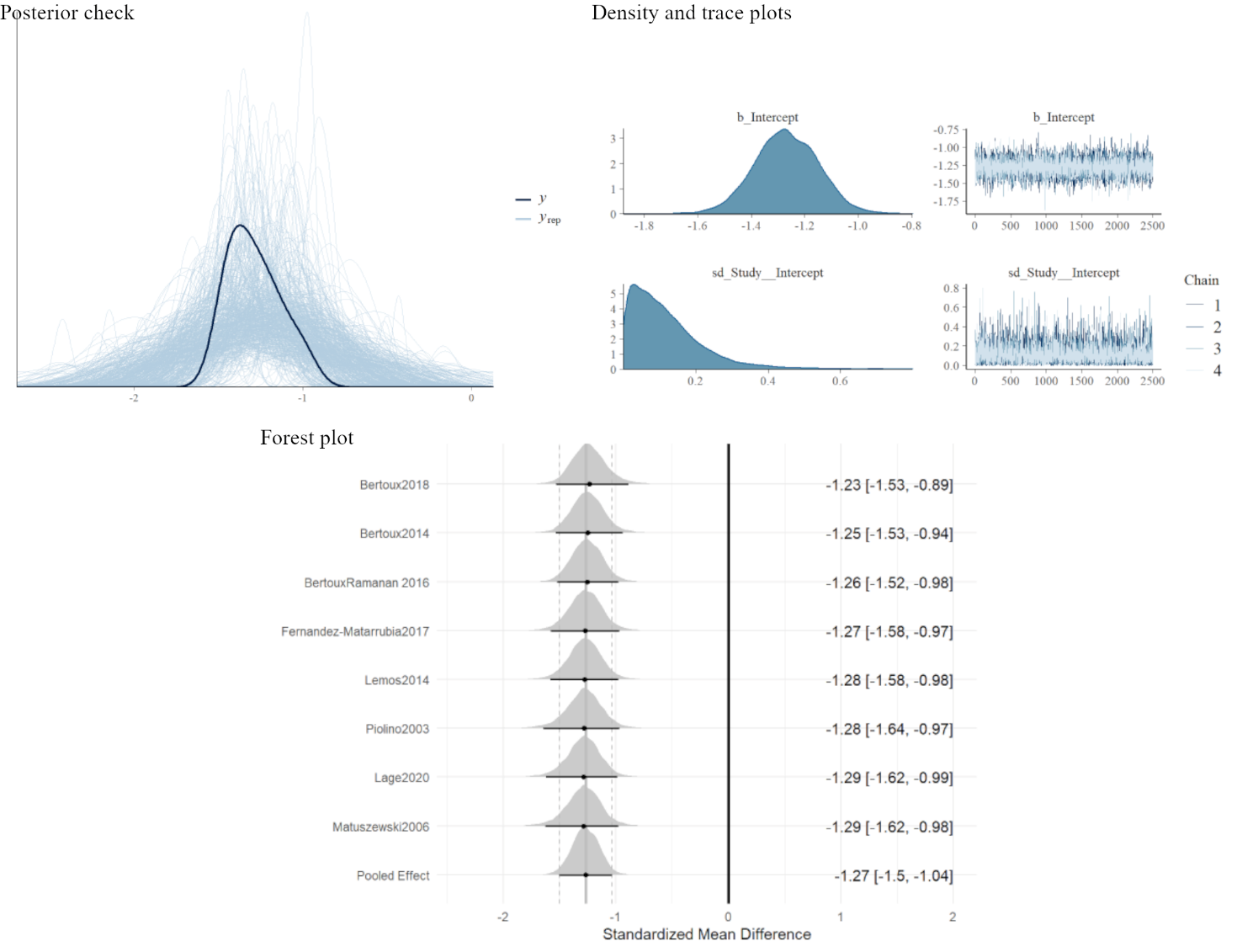


b)
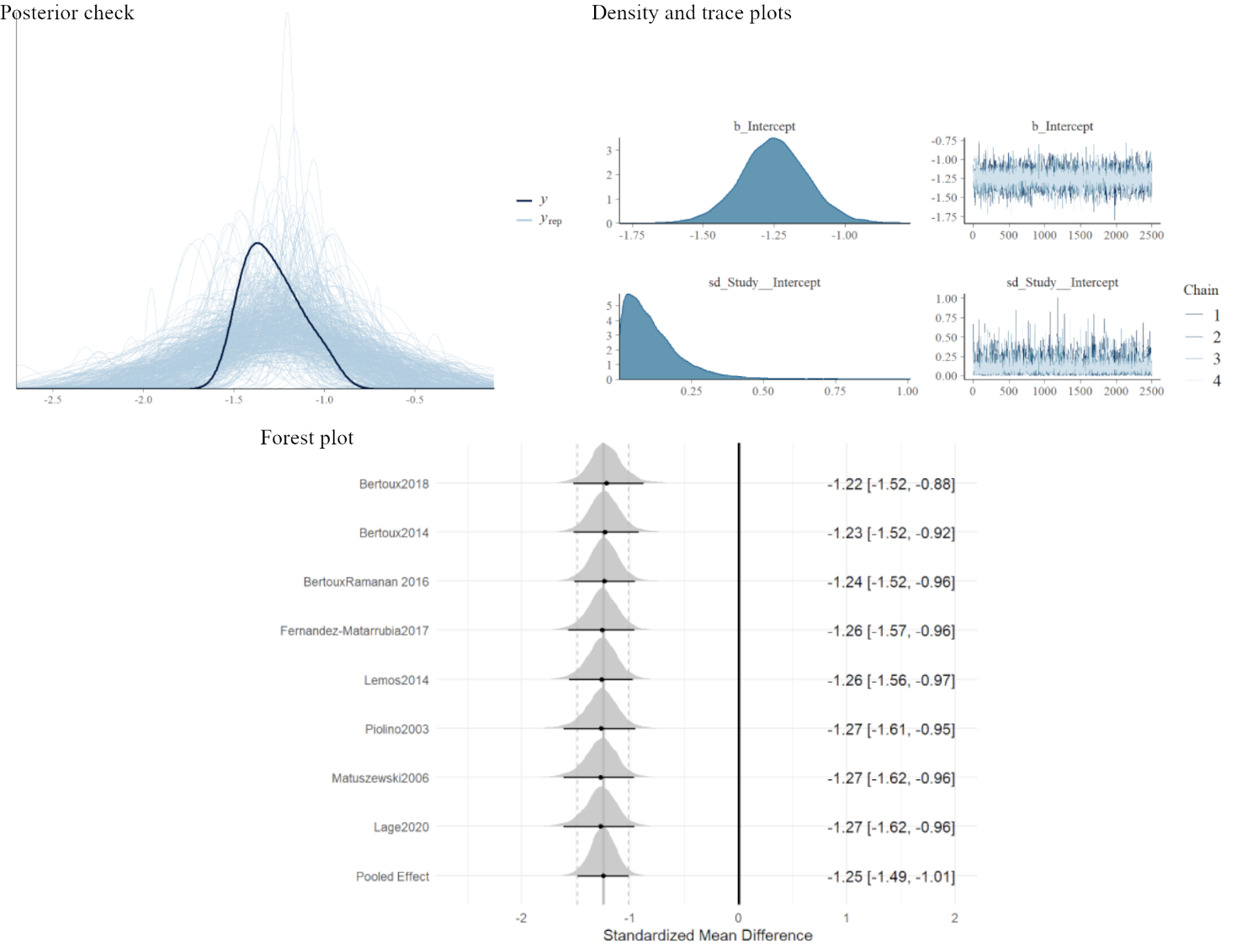


*Free Delayed Recall*

a)
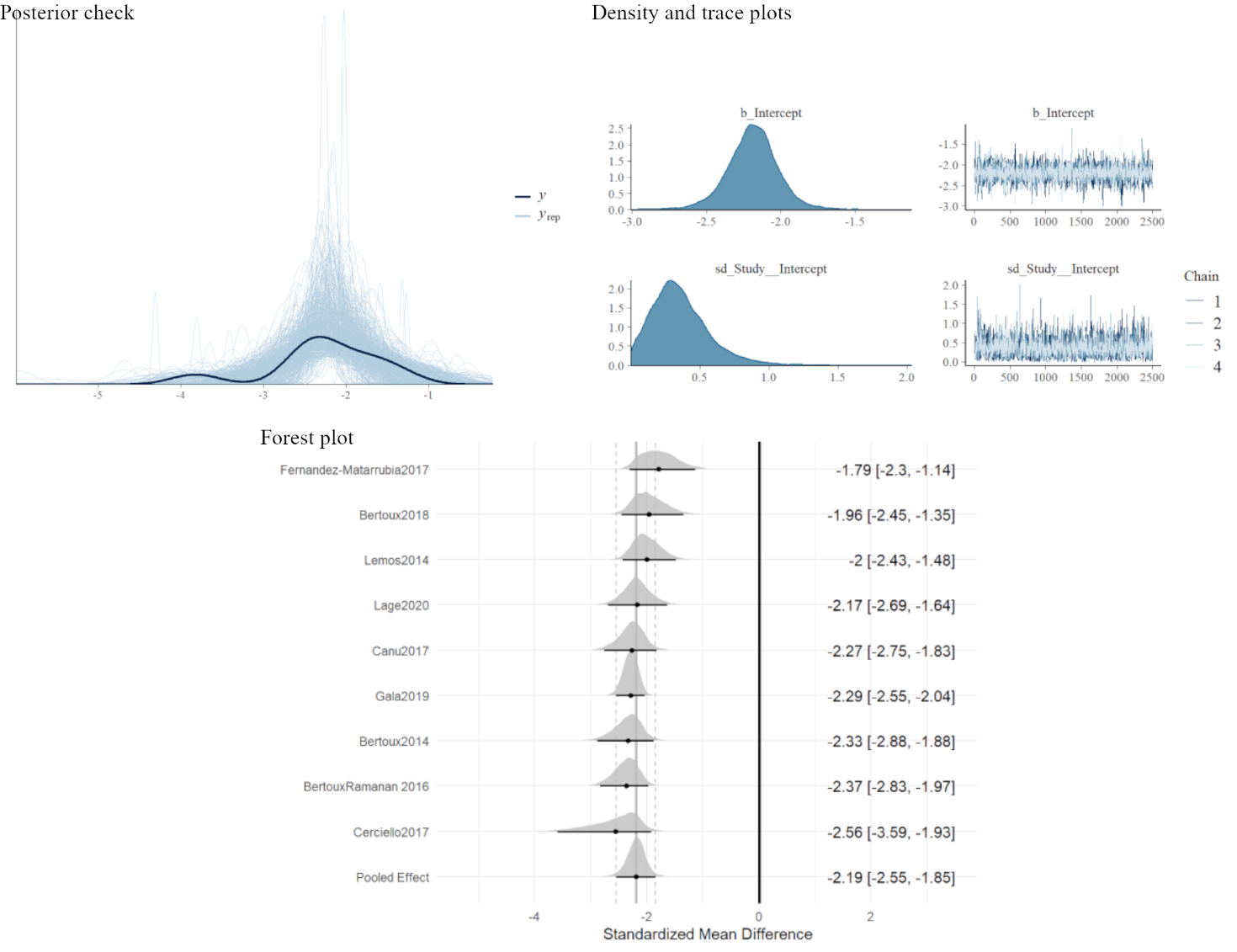


b)
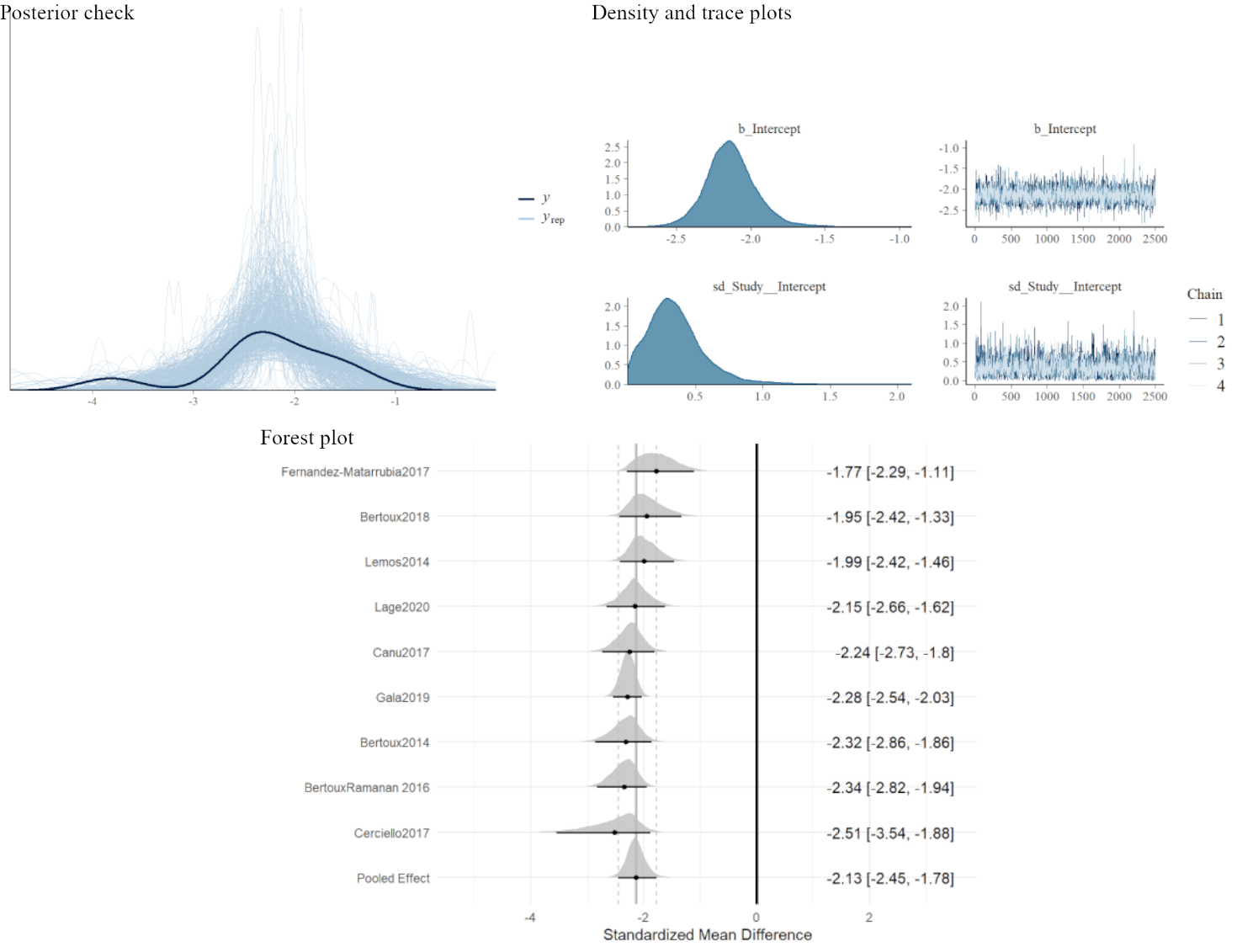


*Total Delayed Recall*

a)
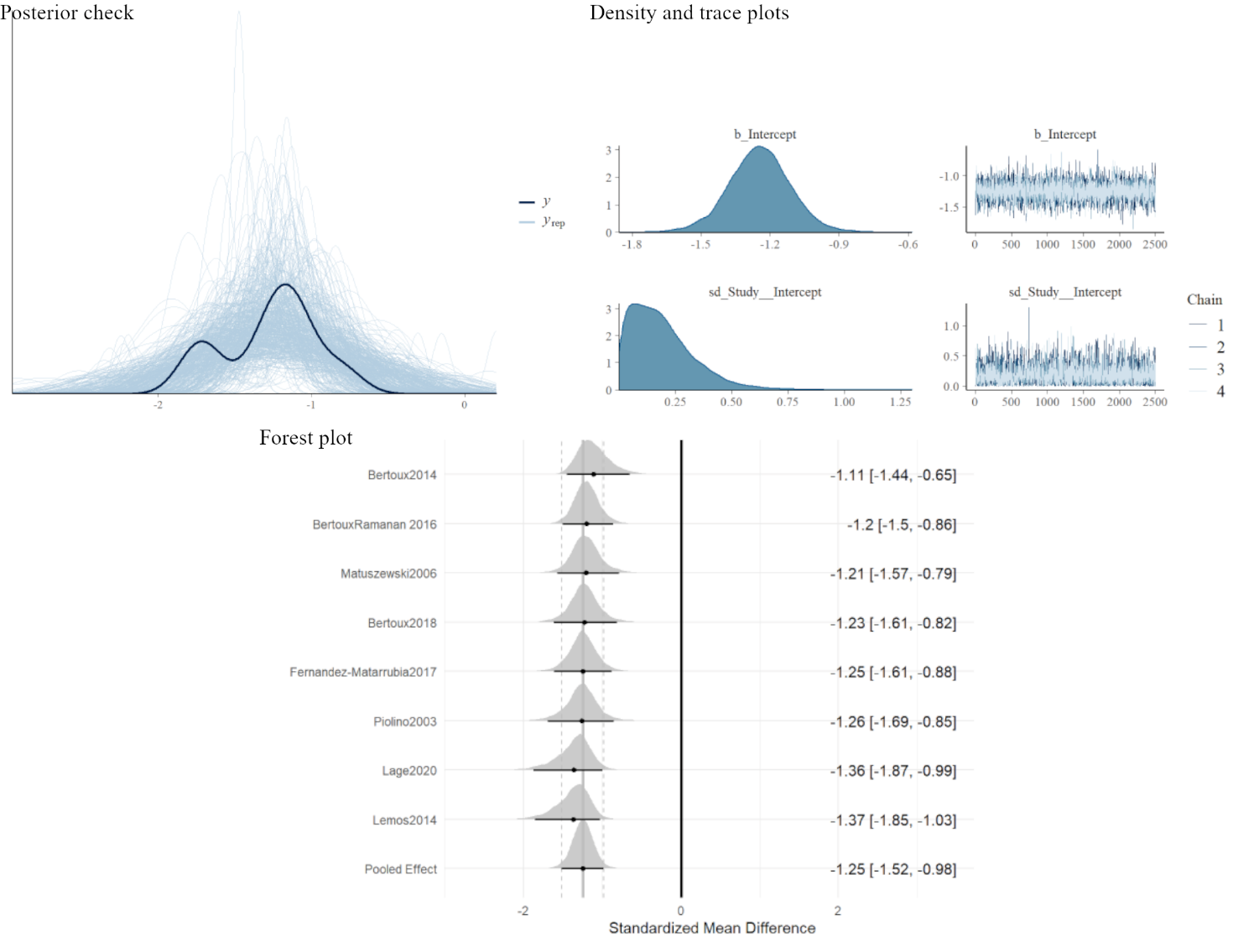


b)
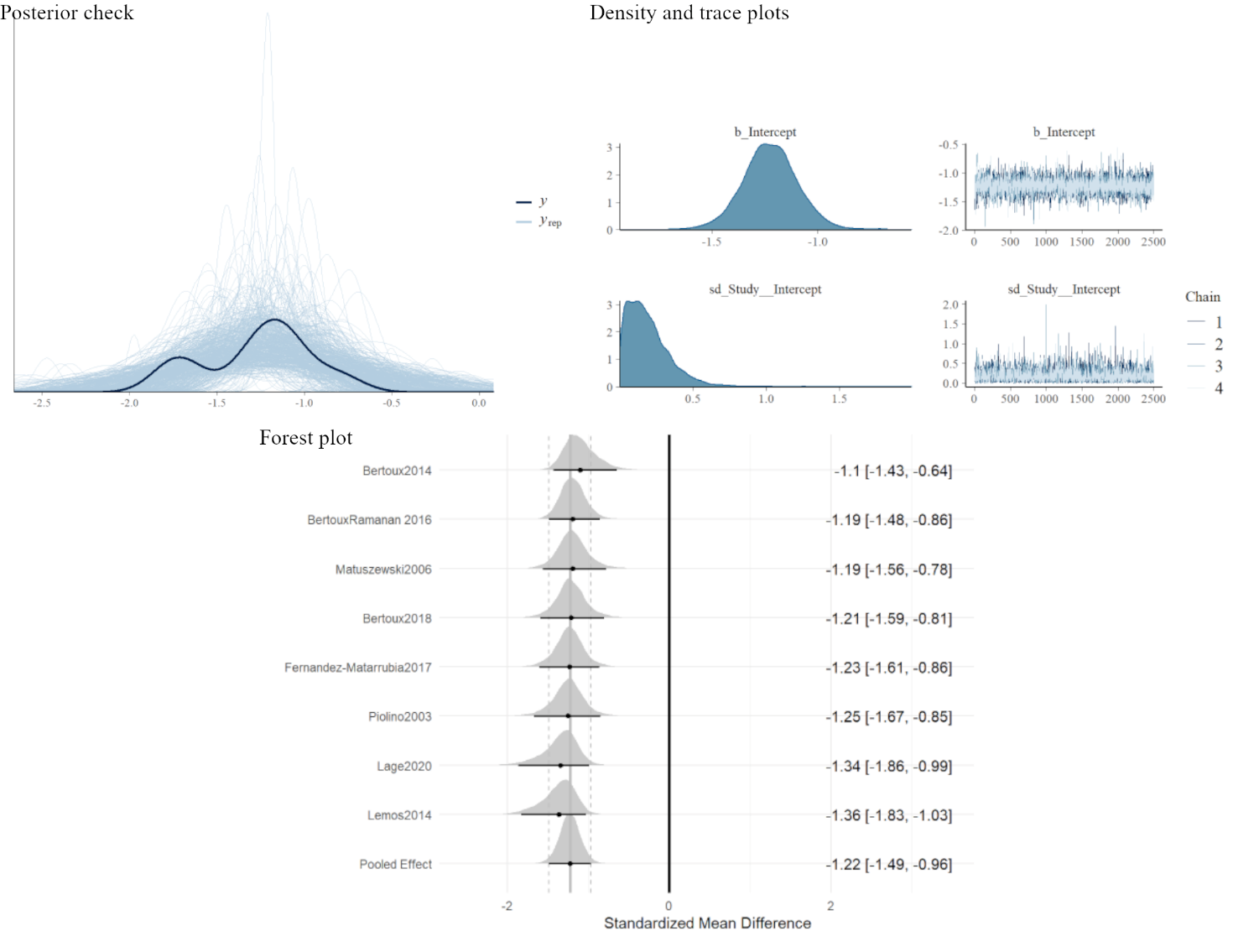


- 1. *bvFTD vs AD*

*Free Immediate Recall*

a)
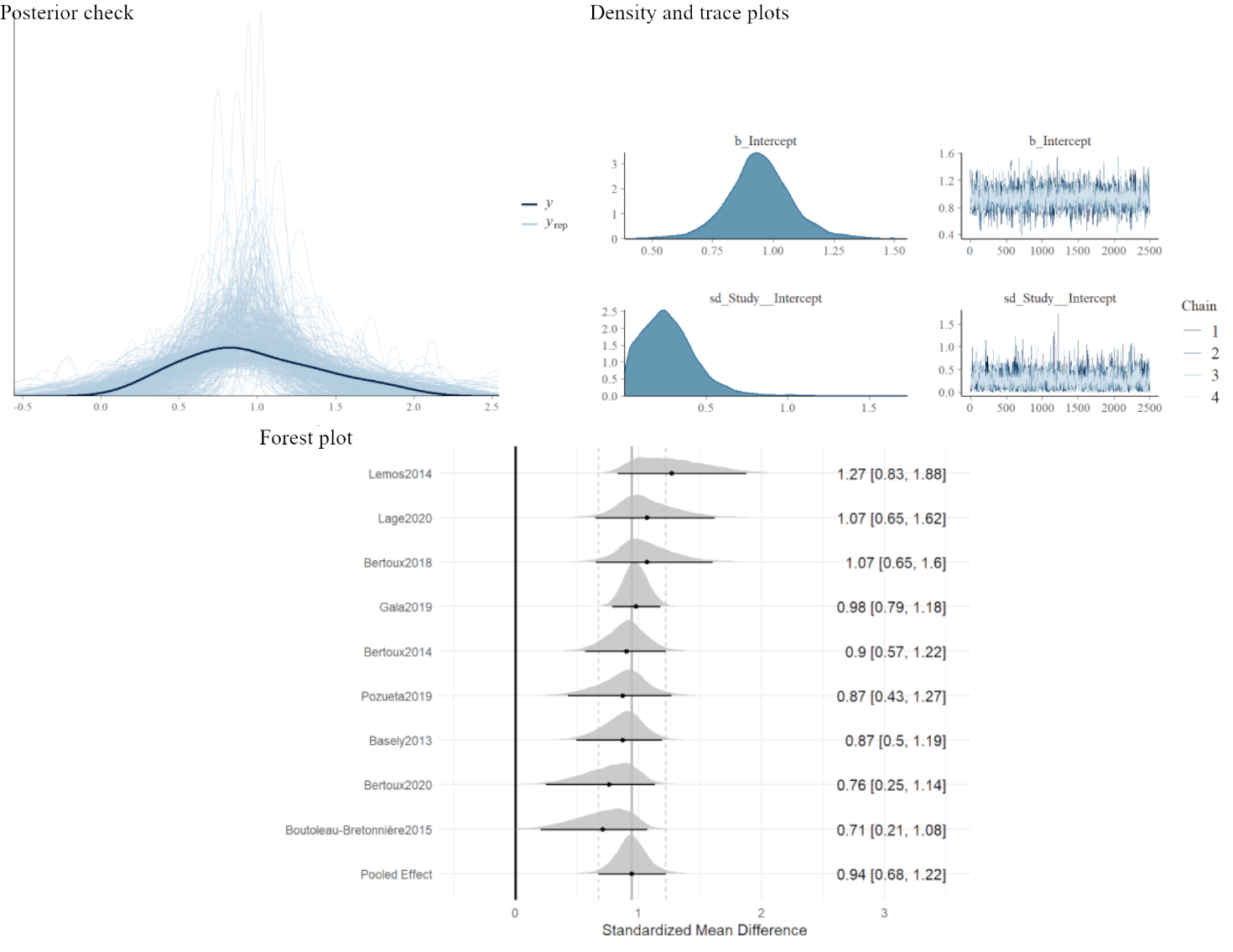


b)
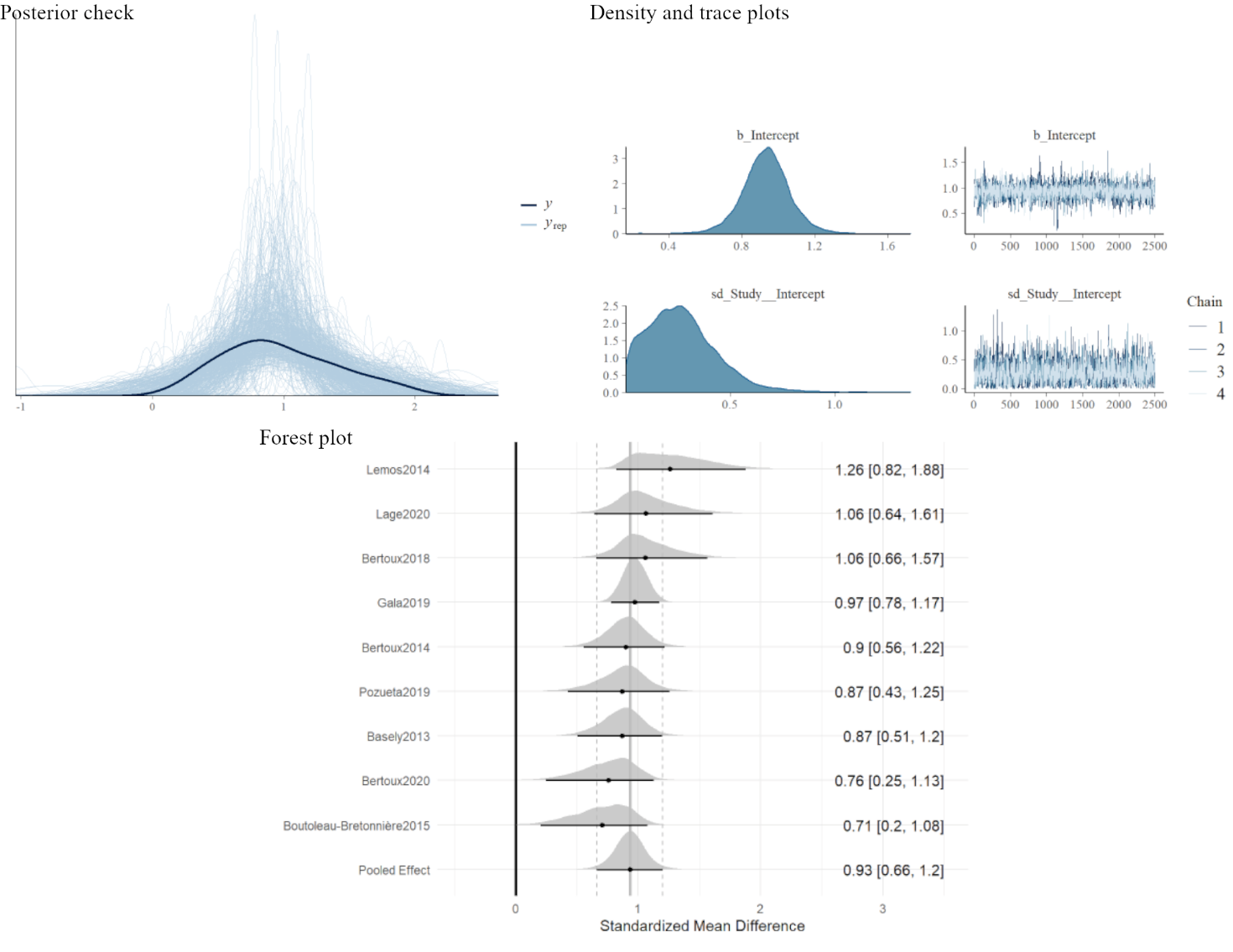


*Total Immediate Recall*

a)
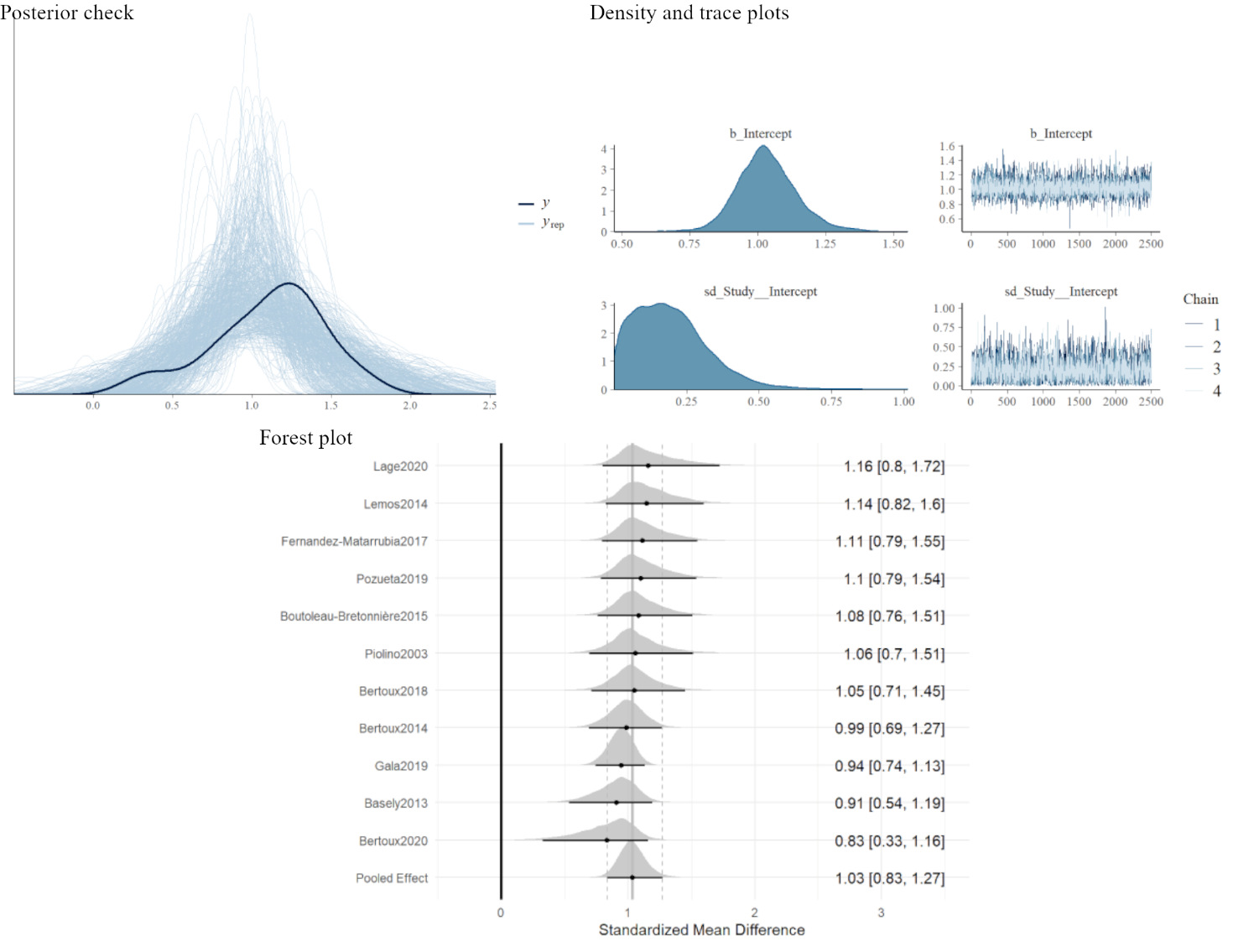


b)*
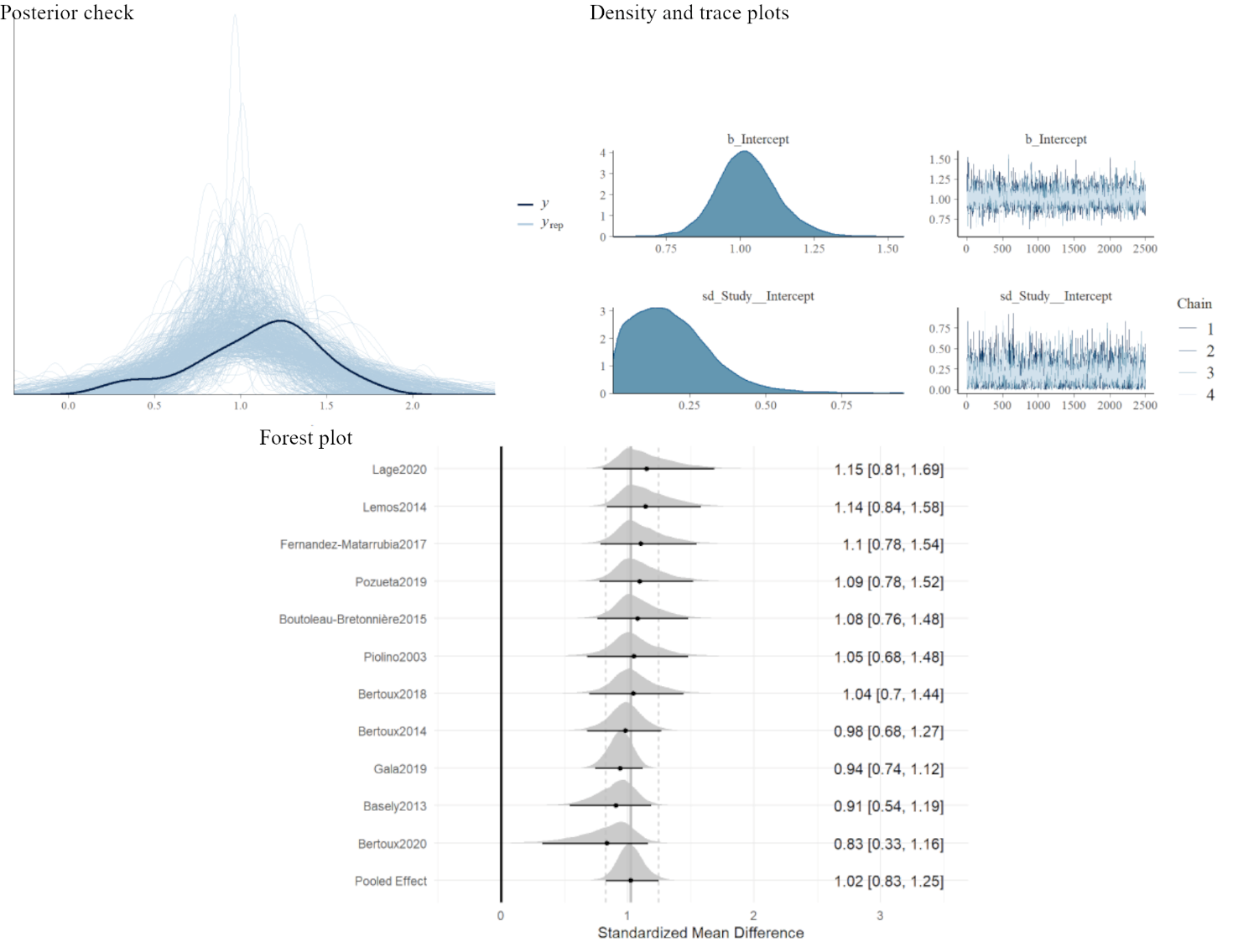
*

*Free Delayed Recall*

a)
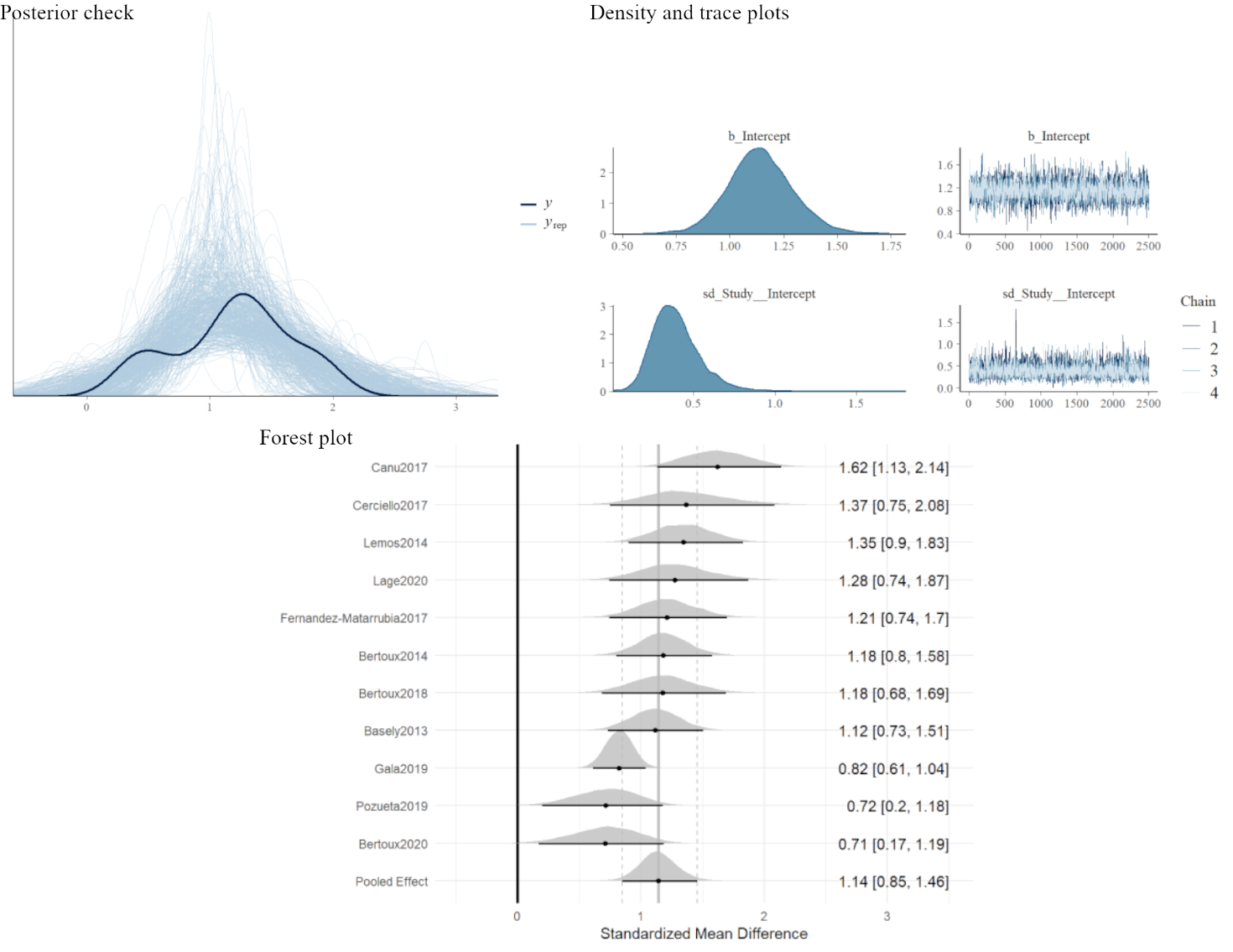


b)*
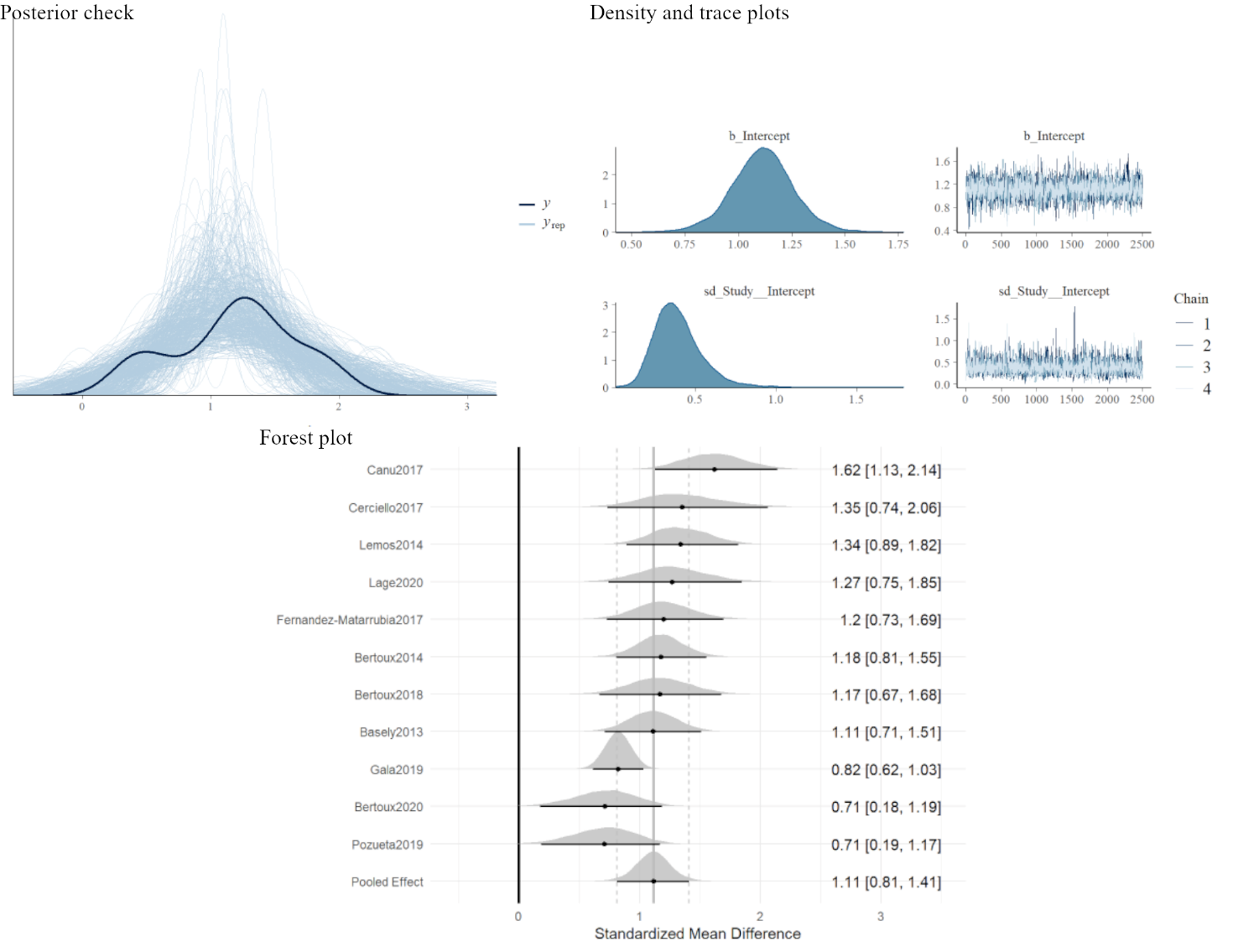
*

*Total Delayed Recall*

a)
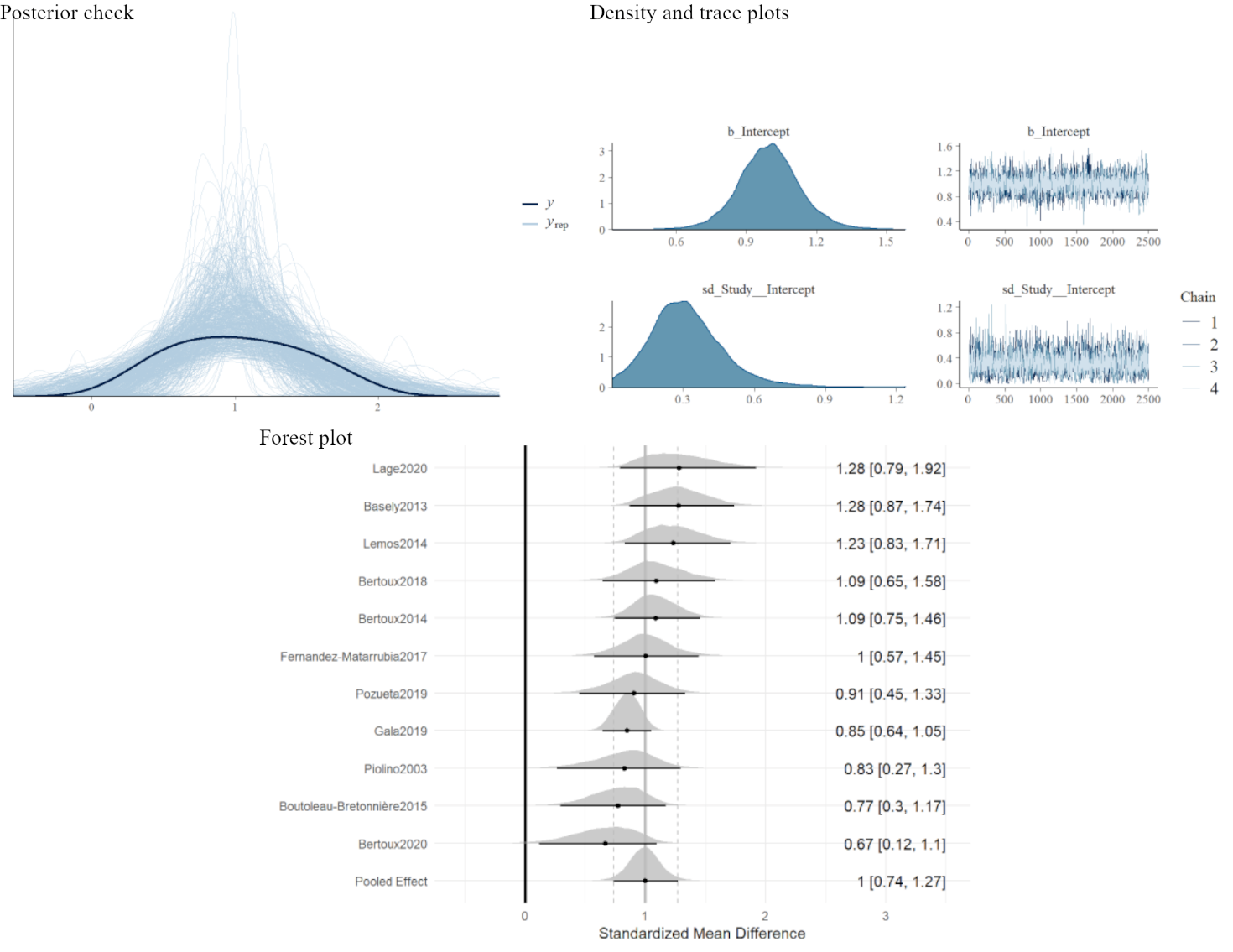


b)
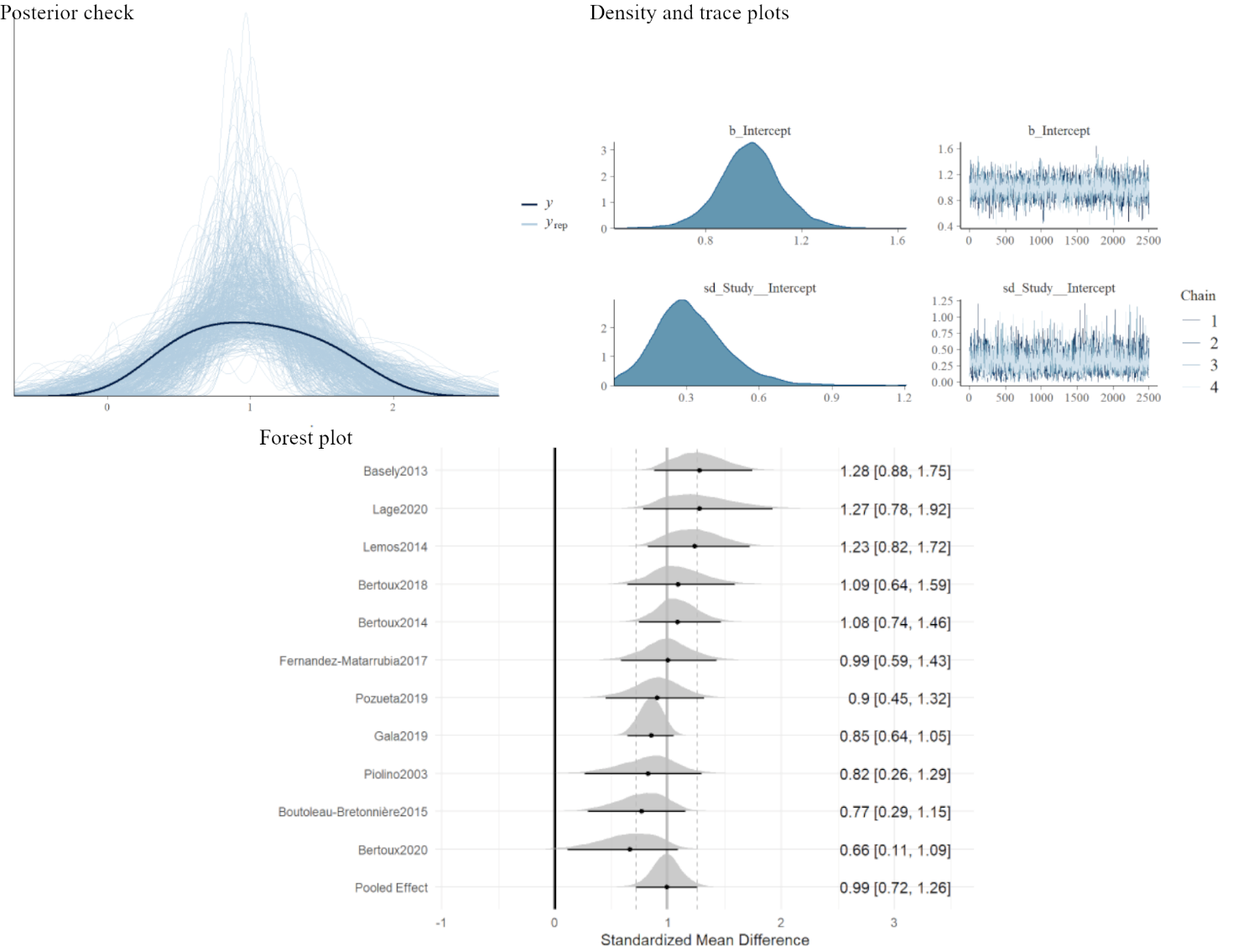


1. **California Verbal Learning Test systematic review**

On 11/28/2022, we searched four databases (PMC, Scopus, Web of Science, and PubMed) using the following search string: ("frontotemporal dementia" OR "frontal dementia" OR "Pick's disease" OR "frontotemporal lobe dementia" OR "frontal lobe dementia" OR "dementia of the frontal type" OR "behavioral variant frontotemporal dementia" OR "bvFTD") AND ("California verbal learning test" OR "CVLT").

We examined the presence of possible duplicates in the obtained pool of records using the R package "Revtools", screening for identical titles or DOIs. Titles and abstracts were analysed, and potentially eligible papers were collected in full text. Each abstract was investigated independently by two authors. In the case of disagreement, the authors' team reviewed the record. The study selection followed the same inclusion criteria defined for the FCSRT (see manuscript). Thirteen studies were included in the systematic review.

Each eligible full-text article was analysed independently by two reviewers of the authors' team to retrieve the following sub-scores in the FCSRT: Free Immediate Recall (total free recall), Free Delayed Recall (long-delay free recall), and Cue Delayed Recall (long-delay cued recall).

One paper reported the Free Delayed Recall scores; one paper reported the Free Immediate Recall and the Free Delayed Recall scores. Eleven papers did not report any data needed for the meta-analysis, so we asked the corresponding author for their dataset. We completed three attempts before excluding the paper. None of the contacted authors provided the requested data.

Thus, the final pull of papers included only two studies and four contrasts. Given the data reported in the papers, we would have added two data points to the Free Delayed Recall analysis and one to the Free Immediate Recall analysis.

A PRISMA Flow Diagram (Page et al., 2021) is included to show the search procedure.

**Identification of studies via databases and registers**

**Records identified through database searching**

**(n = 62)**

PubMed (n = 5)

PMC (n = 40)

Scopus (n = 15)

Web of Science (n = 2)

**Records identified through other sources (n = 7)**

**Identification**

**Records removed *before screening*:**

Duplicate records removed (n = 7)

Records screened

(n = 62)

Records excluded (n = 28)

Reports not retrieved (n = 1)

Reports sought for retrieval

(n = 34)

**Screening**

Reports excluded (n = 19):

Studies not based on CVLT (n = 2)

Studies not concerning bvFTD (n = 3)

Studies without control group (n = 6)

Repeated sample (n = 1)

Sample < 10 (n = 4)

Studies before 1998 (n = 1)

Other reasons (n = 2)

Data requested (n = 14):

Missing dataset requested (n = 12)

Missing sub-scores requested (n = 2)

**Included**

Studies included in meta-analysis

(n = 0)

Studies included in review

(n = 14)

Reports assessed for eligibility

(n = 33)
